# ENSO Modulations due to Interannual Variability of Freshwater Forcing and Ocean Biology-induced Heating in the Tropical Pacific

**DOI:** 10.1038/srep18506

**Published:** 2015-12-18

**Authors:** Rong-Hua Zhang, Chuan Gao, Xianbiao Kang, Hai Zhi, Zhanggui Wang, Licheng Feng

**Affiliations:** 1 Key Laboratory of Ocean Circulation and Waves, Institute of Oceanology, Chinese Academy of Sciences, Qingdao, China; 2 University of Chinese Academy of Sciences, Beijing, 100029, China; 3 National Marine Environmental Forecasting Center, State Oceanic Administration, Beijing 100081, China; 4 College of Atmospheric Sciences, Nanjing University of Information Science and Technology, Nanjing, 210044, China

## Abstract

Recent studies have identified clear climate feedbacks associated with interannual variations in freshwater forcing (FWF) and ocean biology-induced heating (OBH) in the tropical Pacific. The interrelationships among the related anomaly fields are analyzed using hybrid coupled model (HCM) simulations to illustrate their combined roles in modulating the El Niño-Southern Oscillation (ENSO). The HCM-based supporting experiments are performed to isolate the related feedbacks, with interannually varying FWF and OBH being represented individually or collectively, which allows their effects to be examined in a clear way. It is demonstrated that the interannual freshwater forcing enhances ENSO variability and slightly prolongs the simulated ENSO period, while the interannual OBH reduces ENSO variability and slightly shortens the ENSO period, with their feedback effects tending to counteract each other.

The El Niño-Southern Oscillation (ENSO) is the dominant mode of interannual variability with global influence. ENSO originates from air-sea interactions among the sea surface temperature (SST), surface winds, and the thermocline within the tropical Pacific (i.e., the Bjerknes feedback^1^). The ENSO has been seen to exhibit significant modulations in its properties, including its amplitude and time scales^2^,^3^. Although remarkable progress has been made in ENSO studies over the past several decades, the mechanisms for the modulations of the ENSO remain elusive.

Numerous studies have identified roles played by various forcings and feedbacks in ENSO processes. For example, freshwater flux is one important atmospheric forcing to the ocean. In the tropical Pacific, large interannual fluctuations in precipitation (P) and freshwater flux (here focused on precipitation minus evaporation, P-E) have been well documented in association with the ENSO (Supplementary section). They directly affect sea surface salinity (SSS), the depth of the mixed layer (MLD, H_m_), the buoyancy flux (Q_B_) and the thickness of the barrier layer^4^,^5^. Through affecting the related ocean processes, freshwater flux acts to modulate SST and the ENSO in a significant way^6^,^7^,^8^,^9^.

In addition to this physical process, ocean biology-induced heating (OBH) effects in the tropical Pacific have recently received attention due to its pronounced biological responses to the ENSO^10^. The large interannual variations of phytoplankton biomass observed in association with the ENSO in the tropical Pacific can induce significant bio-feedbacks on ENSO, with the potential to modulate it. Indeed, satellite data have revealed coherent bio-climate interactions over the tropical Pacific^11^. In particular, diagnostic analyses and focused modeling studies have demonstrated that ocean biology can affect seasonal and interannual climate variability in the tropical Pacific through its influence on the vertical penetration of sunlight in the upper ocean^12^,^13^,^14^,^15^,^16^,^17^,^18^,^19^,^20^,^21^,^22^,^23^. The penetration depth (H_p_) of solar radiation in the vertical has been introduced to represent the biology-mediated solar radiation uptake by the upper ocean^24^.

Currently, there exists large uncertainty in ways to represent the FWF and OBH effects in climate models, with various approximations often being made. For example, in many Coupled Model Intercomparison Project phase 5 (CMIP5) simulations, interannually varying OBH effects are not adequately taken into account because the chlorophyll (Chl) field in models is often prescribed as seasonally varying climatology only. Additionally, current climate simulations exhibit systematic model biases over the tropical Pacific. For example, the so-called double ITCZ (intertropical convergence zone; an indication of overestimated precipitation) is still a pronounced model bias over the tropical Pacific^25^. Previously, the effects of FWF- and OBH-related feedbacks on the ENSO have been separately examined using ocean-atmosphere models^8^,^21^. It is found that ENSO simulations are sensitively dependent on the way each individual feedback is represented. Because these two processes are interrelated within the tropical Pacific climate system, understanding how the ENSO is collectively modulated by their influences is critically important to short-term ENSO forecasts and the long-term projection of ENSO changes in the global warming context^3^,^26^.

Here, as a demonstration, we investigate how the combined effects of these two feedbacks can be clear sources for ENSO modulations and biases using a hybrid coupled model (HCM), which consists of an ocean general circulation model (OGCM) and an empirical model for interannual wind stress anomalies (the details of the HCM can be found in Zhang *et al.*^21^,^27^, Zhang and Busalacchi^8^, and in the Supplementary section). As shown in these previous modeling studies, the HCM has an ability to capture the main physical feedbacks (e.g., the Bjerknes feedback involving interactions among the SST, surface wind and thermocline, and zonal advective feedback) so that the ENSO cycles are produced within the coupled ocean-atmosphere system of the tropical Pacific. To further represent the related FWF and OBH feedback processes, satellite data are used to construct empirical models for interannually varying freshwater flux and H_p_ fields in the tropical Pacific (Supplementary section). Then, these empirical models are incorporated into the HCM. To isolate the feedback effects associated with interannually varying FWF and OBH, four experiments are performed (see Methods). In a reference run denoted as FWF_inter_-OBH_inter_, interannual anomalies of freshwater flux and H_p_ are both determined using their corresponding empirical models to take into account the related feedbacks. Other HCM-based experiments are further conducted in which FWF and OBH are alternatively taken as interannually and/or climatologically varying, allowing their feedback effects to be examined separately or collectively in a clear way.

## Results

**Figure 1:**
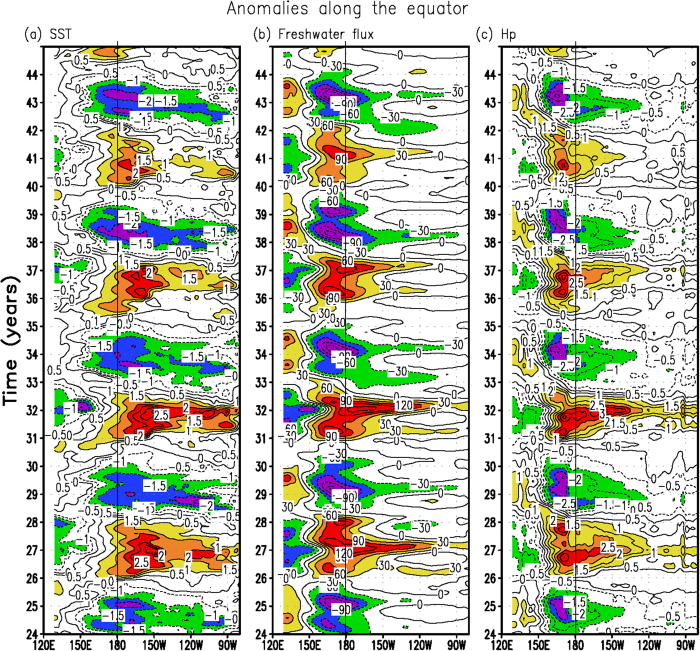
Interannual anomaly fields along the equator simulated from the reference run (FWF_inter_-OBH_inter_): (a) SST, (b) freshwater flux, and (c) H_p_. The HCM was integrated for more than 100 years and the plotting is shown only from model year 24 to 44 for clarity. The contour interval is 0.5 °C in (**a**), 30 mm month^−1^ in (**b**), and 0.5 meter in (**c**).

**Figure 2:**
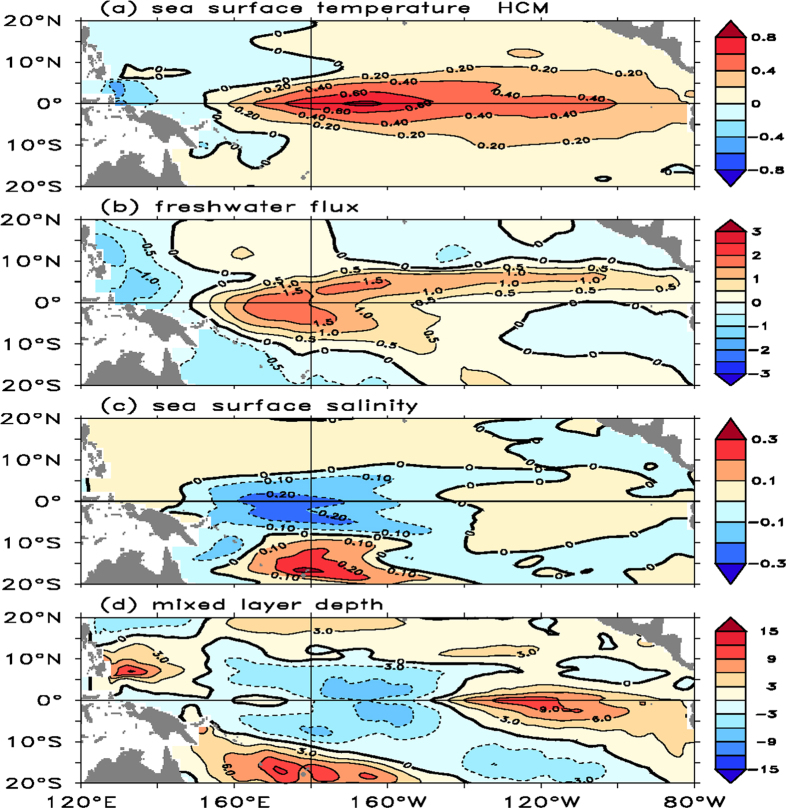
Spatial patterns of the first EOF SST mode and the corresponding regression patterns of some related anomaly fields with the principal component (PC) of the first EOF SST mode obtained from the reference HCM run (FWF_inter_-OBH_inter_): (a) SST, (b) FWF, (c) SSS, and (d) MLD. The EOF analyses are performed using interannual SST anomalies simulated from the HCM during model year 24 and 54. The units are °C for SST, mm day^−1^ for FWF, psu for SSS and m for MLD. The corresponding results analyzed from ARGO and from the GFDL ESM2M are shown in the Supplementary section.

Examples of simulated fields from the reference run (FWF_inter_-OBH_inter_) are shown in Fig. 1. It is evident that the HCM can quite well depict the mean ocean climatology and its variability in the tropical Pacific compared with observations^28^ (Some corresponding observations and model validations are presented in the Supplementary section; a quantitative comparison between observations and simulations is quantified in Table 1 in terms of the standard deviations of interannual anomalies). For example, the coupled model captures a pronounced interannual oscillation, with a dominant standing pattern of SST variability on the equator (Fig. 1a). Accompanied with ENSO-induced SST anomalies, the freshwater flux (Fig. 1b) and H_p_ (Fig. 1c) exhibit large interannual variations over the western-central equatorial Pacific. Because the HCM does not include stochastic atmospheric forcing^24^, the ENSO simulated is quite regular, with similar space-time evolutions from one event to another. However, the model to some extent also exhibits the ENSO diversity in each event (e.g. its amplitude, frequency and period, and the eastern Pacific (EP) type El Niño vs. the central Pacific (CP) type El Niño). To generalize common features of the spatial pattern and their relationships for El Niño and La Niña events, we perform a composite analysis by adopting empirical orthogonal function (EOF) and regression methods. Figure 2 shows the results obtained from the HCM simulation; the corresponding results analyzed from ARGO and from the GFDL ESM2M (the GFDL Earth System Model, which is a CMIP5-type model) are shown in the Supplementary section. Note that the 1st EOF SST mode of the HCM indicates the CP type of El Niño (Fig. 2a), whereas the observed SST displays the canonical El Niño. Thus, the HCM exhibits the lack of variability in the Cold Tongue region where the bio-physical feedback is expected to be the strongest^13^. The disagreement between the HCM simulations and observations may influence the conclusion derived. However, the spatial patterns of these related anomaly fields and their relationships simulated from the HCM match well with corresponding observations and the GFDL ESM2M-based analyses. In particular, the patterns of SSS and MLD simulated from the HCM (Fig. 2c,d) look similar to those observed. This indicates that our simplified HCM is doing reasonably well in simulating ENSO evolution relative to observations and coupled general circulation model (CGCM) simulations. Although the simplified coupled model actually has bias, it may capture some essential feedbacks.

Coherent patterns and interrelationships are seen among these anomaly fields. In particular, interannual variations in freshwater flux and H_p_ represent not only a response to the ENSO, but also a feedback onto the ENSO. For example, El Niño conditions (as represented in Fig. 2 and in the Supplementary section) are characterized by a positive SST anomaly in the central-eastern equatorial regions, accompanied with a positive freshwater flux anomaly (the freshwater flux is into the ocean; Fig. 2b), a negative SSS anomaly (Fig. 2c) and a shallow ML (Fig. 2d) over the western-central equatorial Pacific (Some observed space-time features of these anomaly fields are illustrated in the Supplementary section). A corresponding feedback loop can be traced in association with the interannually varying FWF^8^. The positive freshwater flux anomaly acts to enhance the negative SSS anomaly, which decreases surface density in the western-central equatorial basin. The induced changes in density (less dense in the mixed layer) act to enhance the stratification and stabilize the upper ocean, leading to a decrease in the vertical mixing. Additionally, the positive freshwater flux anomaly tends to increase a negative buoyancy flux (*Q*_B_) anomaly with a shallower ML (Fig. 2d), which acts to weaken the entrainment of subsurface waters into the mixed layer. These freshwater-induced ocean processes exert a direct influence on SST such that El Niño events are enhanced by the positive freshwater flux anomaly (Fig. 2b), leading to a positive feedback onto the ENSO.

**Figure 3:**
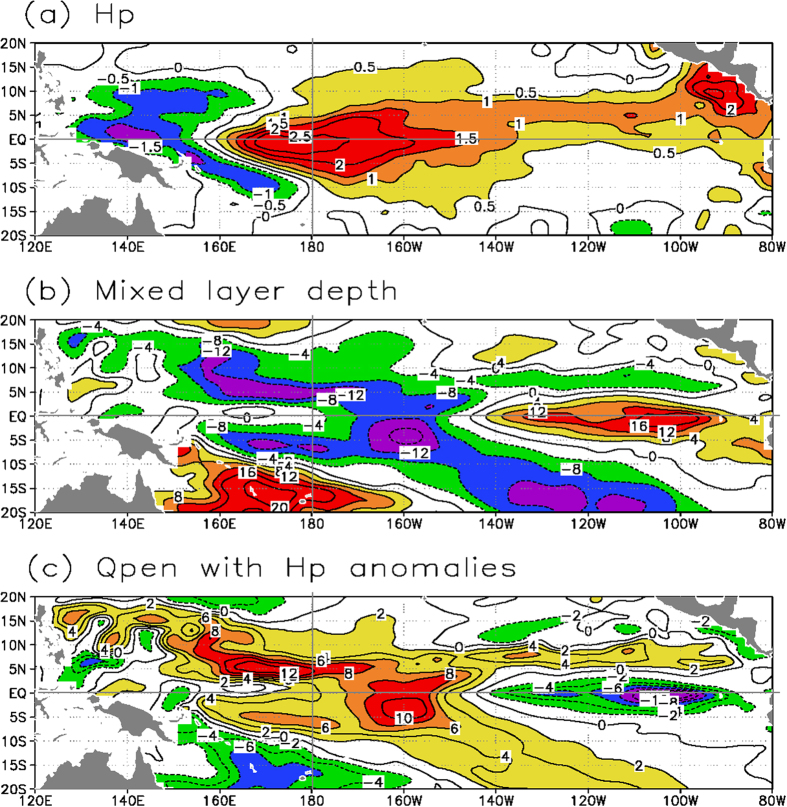
Horizontal patterns of interannual anomalies simulated from the reference run (FWF_inter_-OBH_inter_) for El Niño conditions as represented in August of model year 31: (a) H_p_, (b) H_m_, and (c) Q_pen_. Here Q_pen_ is denoted as the penetrative solar radiation flux out of the bottom of the mixed layer, written as 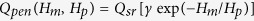 where Q_sr_ is the incoming solar radiation flux at the sea surface, H_m_ is the mixed layer depth, H_p_ is the penetration depth of solar radiation, γ is a constant (=0.33) which denotes the fraction of solar radiation that is available to penetrate to depths beyond the first few centimeters of the sea surface. The contour interval is 0.5 meter in (**a**), 4 meters in (**b**), and 2 W m^−2^ in (**c**).

On the OBH effect side, the El Niño condition is accompanied with decreased ocean biology activity/productivity over the equatorial Pacific, as clearly indicated by a positive H_p_ anomaly (Figs 1c and 3a). Correspondingly, the H_m_ anomaly is negative in the western-central equatorial Pacific (Fig. 3b), accompanied by a positive Q_pen_ anomaly (Fig. 3c; Q_pen_ is the penetrative solar radiation flux throughout the bottom of the ML as detailed in the Supplementary section). Note that the interannual variability of H_m_ is a major factor determining that of Q_pen_ because the spatial pattern of the former is nearly mirrored by that of the latter (i.e., during El Niño, the shallow ML in the western-central equatorial Pacific causes solar radiation that is trapped less directly within the ML but penetrated more out of the bottom of the ML). However, ocean biology variability (as indicated by H_p_) can also make a significant contribution to interannual Q_pen_ variability (Fig. 3c) over the western-central equatorial Pacific, where large interannual anomalies of H_p_ (Fig. 3a), as a response to the ENSO, tend to be out-of-phase with those of H_m_ (Fig. 3b). As a result, the effect of the interannual variability of H_p_ on Q_pen_ over the western-central equatorial Pacific can be comparable with that of H_m_ during ENSO cycles. A corresponding feedback loop can be traced in association with interannual variations in H_p_ 21. During El Niño, the H_p_ anomaly is positive in the western-central equatorial Pacific (Fig. 3a), acting to significantly enhance the positive Q_pen_ anomaly in the region (Fig. 3c; Supplementary section), with the sunlight vertically penetrating more throughout the bottom of the ML and being trapped less directly within the ML. Thus, the ocean biology-mediated solar radiation uptake leads to a decrease in the vertical temperature contrast during El Niño (less direct heating in the ML but more direct heating in the subsurface layers). The induced differential heating in the vertical acts to weaken the stratification and de-stabilize the upper ocean, with increases in the vertical mixing and entrainment of subsurface waters into the ML. Thus, the OBH is seen to alter ocean processes and exert an influence on SST such that the intensity of El Niño events is reduced, leading to a negative feedback onto the ENSO.

Note that various forcings and feedbacks are included within the tropical Pacific climate system (e.g., the Bjerknes feedback and those associated with FWF and OBH), collectively shaping the ENSO properties as evident in the reference run (Fig. 1). In particular, as a focus in this study, large interannual variations in FWF and OBH are seen in the western-central equatorial Pacific, representing not only a response to ENSO but also a feedback onto ENSO. As analyzed above, these two feedbacks tend to modulate dynamic processes in the upper ocean (i.e., the ocean density and vertical mixing), which can exert an influence on SST, a field that directly induces changes to the atmosphere. The interrelationships among the related interannual anomalies in the reference run indicate possibility of their combined effects that can be induced by the interannually varying FWF and OBH feedbacks. However, the nature of their combined effects has not been demonstrated. In addition, these two processes are interactively represented in the reference run, with their combined roles being lumped together, making it difficult to clearly illustrate their modulating effects on the ENSO.

**Figure 4:**
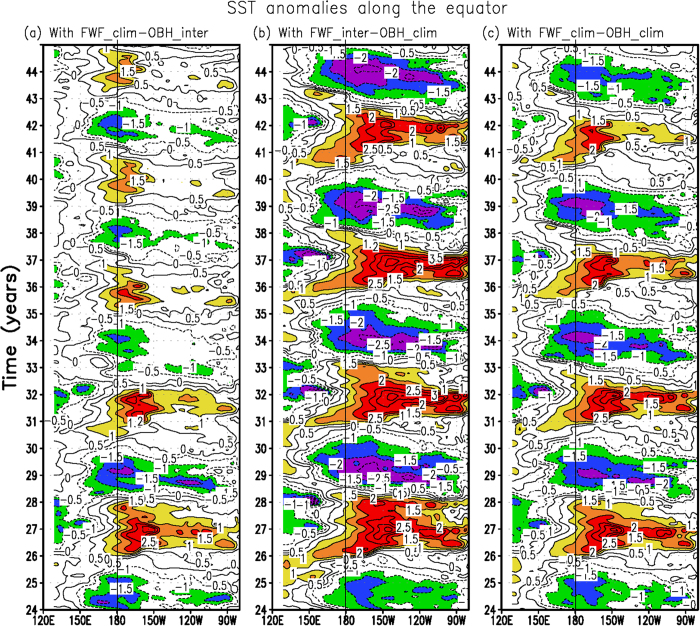
Interannual SST anomalies along the equator simulated in (a) FWF_clim_-OBH_inter_, (b) FWF_inter_-OBH_clim_, and (c) FWF_clim_-OBH_clim_. The contour interval is 0.5 °C.

**Figure 5:**
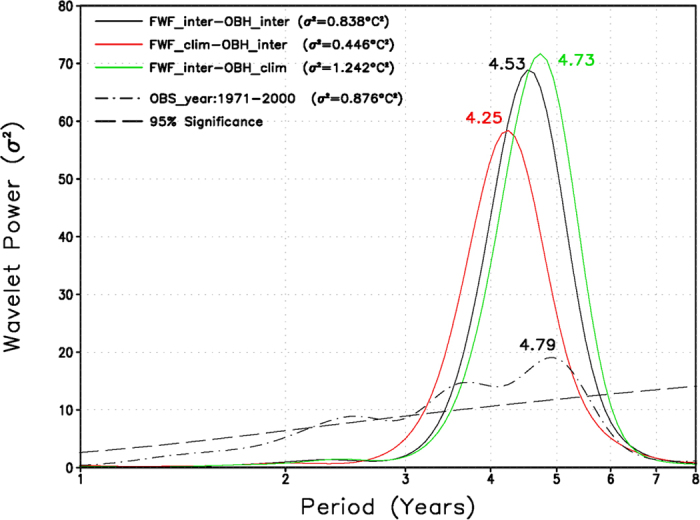
Wavelet power spectra for Niño3.4 SST anomalies calculated in FWF_inter_-OBH_inter_, FWF_clim_-OBH_inter_, and FWF_inter_-OBH_clim_. The observed SST data used for the corresponding calculation are from Smith *et al.* (2008). The dot-dashed line is the 95% significance level for these runs, assuming a white noise process.

To clearly demonstrate the individual effect and to support the above diagnosis-based arguments, three more HCM experiments are performed (Method section); the simulated interannual SST anomalies along the equator are illustrated in Fig. 4. The effects of interannually varying FWF on ENSO are clearly evident when comparing the results in FWF_inter_-OBH_inter_ (Fig. 1a) with those in FWF_clim_-OBH_inter_ (Fig. 4a). When the interannually varying FWF effect is disabled in FWF_clim_-OBH_inter_, a significant modulating effect emerges on the ENSO amplitude and time scales. As seen in Figs 1, 4 and 5, the interannual variability in FWF_clim_-OBH_inter_ is significantly weakened. The effects are further quantified in Table 1. For example, the standard deviations of the Niño3 and Niño4 SST anomalies are 0.84 °C and 0.96 °C in FWF_inter_-OBH_inter_ and 0.62 °C and 0.78 °C in FWF_clim_-OBH_inter_, respectively. Comparing the simulations in FWF_inter_-OBH_inter_ with FWF_clim_-OBH_inter_, these values represent a decrease of approximately 26% (19%) for the Niño3 (Niño4) SST variability. Additionally, the standard deviations of zonal wind stress and SSS in the Niño4 region are 0.021 N m^−2^ and 0.19 psu in FWF_inter_-OBH_inter_; they decrease to 0.016 N m^−2^ and 0.13 psu in FWF_clim_-OBH_inter_ (a decrease of 24% and 32% in terms of wind stress and SSS variabilities, respectively). Moreover, the two runs exhibit clear phase differences. For example, there is a tendency for the ENSO time scales to become shorter in FWF_clim_-OBH_inter_ than in FWF_inter_-OBH_inter_. The commonly used Niño3.4 SST series are used to quantify the dominant time scales of the ENSO. As shown in Fig. 5 from a wavelet analysis, the interannual variability has a sharp peak at approximately 4.5 years in FWF_inter_-OBH_inter_, but at approximately 4.3 years in FWF_clim_-OBH_inter_, with a difference of approximately 3 months in the oscillation periods. These results indicate that the freshwater-induced feedback acts to modulate the ENSO amplitude and time scales. The corresponding period and frequency estimated from observed SSTs are also included in Fig. 5 for quantitative comparisons and model validations. Since the analyses for the changes in ENSO rely on one single experiment of one particular model, we perform statistical significance tests for the changes in interannual variability between the different experiments (Supplementary section).

Next, the effects of the interannually varying OBH on the ENSO are also evident when comparing simulations in FWF_inter_-OBH_inter_ (Fig. 1a) with those in FWF_inter_-OBH_clim_ (Fig. 4b). When the interannually varying OBH effect is disabled in FWF_inter_-OBH_clim_, the interannual variability becomes significantly stronger in FWF_inter_-OBH_clim_ (Figs 1a and 4b). The effects are quantified in Table 1. For example, the standard deviation of the Niño3 SST anomalies is 1.02 °C in FWF_inter_-OBH_clim_. Relative to that in FWF_inter_-OBH_inter_, this value represents an increase of approximately 21% in FWF_inter_-OBH_clim_. Additionally, the standard deviation of the Niño4 zonal wind stress is 0.025 N m^−2^ in FWF_inter_-OBH_clim_, representing an increase of 19% compared with that in FWF_inter_-OBH_inter_. Additionally, there is a clear tendency for the ENSO time scales to become longer in FWF_inter_-OBH_clim_ than in FWF_inter_-OBH_inter_. As shown in Fig. 5 from a wavelet analysis, the interannual variability has a peak at 4.5 years in FWF_inter_-OBH_inter_, but at 4.7 years in FWF_inter_-OBH_clim_, with a difference of 2–3 months in oscillation periods. Thus, the bio-feedback is seen to exert a pronounced influence on interannual variability in the HCM simulations.

Finally we turn to FWF_clim_-OBH_clim_ in which the interannually varying FWF and OBH are both disabled. Because the amplifying FWF effect and the damping OBH effect are not taken into account, the intensity of the interannual variability simulated in FWF_clim_-OBH_clim_ can be inferred to be ranging between FWF_clim_-OBH_inter_ and FWF_inter_-OBH_clim_. As confirmed in Fig. 4c and Table 1, the ENSO amplitude and oscillation periods simulated in FWF_clim_-OBH_clim_ are near to those in FWF_inter_-OBH_inter_ (Fig. 1a) in which the two feedbacks tend to exert a counteracting influence on ENSO.

## Discussion

**Figure 6:**
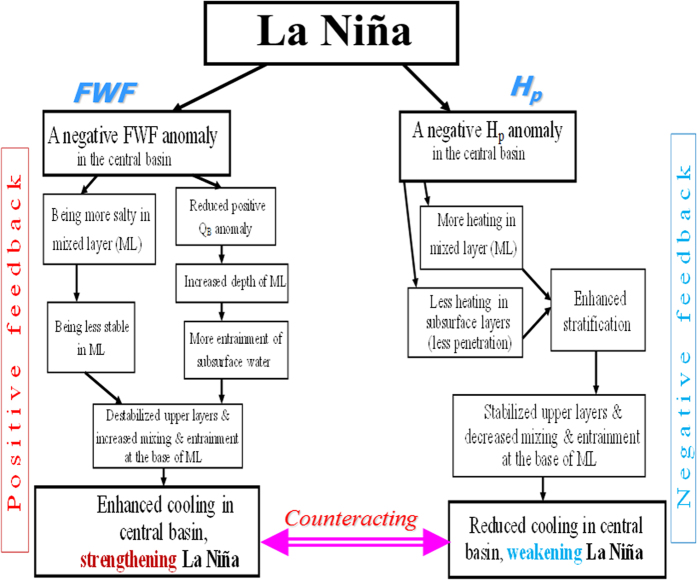
Schematic diagram illustrating the effects induced by FWF and OBH during a La Niña event. The left panel is shown for processes induced by a negative FWF anomaly over the western-central equatorial Pacific, which acts to induce a positive feedback onto the coupled ocean-atmosphere system of the tropical Pacific; the right panel is for processes induced by a negative H_p_ anomaly in the western-central basin, which tends to serve as a negative feedback onto the system.

Climate model simulations in the Intergovernmental Panel on Climate Change Fourth Assessment Reports (IPCC AR4) indicate diverse behaviors in terms of ENSO amplitude and oscillation periods^3^, with no consensus as to future changes in ENSO characteristics. Illustrating ENSO behaviors under complicated background condition changes and the interrelated influences of different feedbacks remain an intensively focused research area. The HCM experiments performed in this study illustrate large modulating effects of FWF and OBH feedbacks on the ENSO. Based on the interrelationships among the related interannual anomalies and sensitivity experiments, the induced effects are summarized in Fig. 6. It is demonstrated that a positive feedback is associated with FWF, which acts to enhance interannual variability and lengthen time scales; by contrast, a negative feedback is associated with OBH, which acts to reduce interannual variability and shorten time scales. Thus, interannually varying FWF and OBH feedbacks can affect ENSO amplitude and time scales in a systematic way. The results from this work obtained using a simplified coupled ocean-atmosphere model have implications for ENSO simulation, prediction and projection in the global warming context.

Various forcings and feedbacks exist in the tropical Pacific and play roles in shaping the interannual variability seen in the HCM simulations, with the ENSO being modulated by their collective effects. For example, in the reference run, the positive feedback effect associated with FWF tends to counteract the negative one associated with OBH. It is thus necessary to include both effects in coupled ocean-atmosphere models in a coherent way. At present, however, uncertainties exist in representing these feedbacks, and various approximations are often made in modeling studies. It follows that if one feedback is included but another is not, model biases can be introduced to ENSO simulations (its amplitude and time scales) as clearly demonstrated using the HCM. For instance, if the interannually varying OBH effect is not included (so that the related damping effect is excluded), the ENSO amplitude can be overestimated as the amplifying effect induced by interannually varying FWF feedback is not adequately balanced by the damping effect associated with OBH. Indeed, Wang *et al.*^29^ found that interannual SST variations simulated in the NOAA CFS (Climate Forecast System) tend to be overestimated; one cause is likely related to the fact that climatology Chl is prescribed in the current CFS (thus, the lack of an interannual damping bio-effect on ENSO). More recently, Kang *et al.*^30^ also found that interannual SST variations are overestimated in simulations using the NCAR CESM1.0 (the Community Earth System Model) in which climatology Chl is prescribed to represent seasonally varying OBH effects only and the interannually varying OBH effect is intentionally disabled. Furthermore, note that there exists the so-called double ITCZ bias in the NOAA CFS and the NCAR CESM1.0, indicating that the FWF effect is not well represented^25^,^29^. Thus, the combination of the lack of interannually varying OBH feedback (the use of prescribed climatological Chl) and the misrepresentation of the interannually varying FWF feedback likely leads to simulated ENSO events in these models that are far too strong^29^,^30^.

In terms of short-term ENSO prediction, there exist large uncertainties and a wide range of real-time ENSO forecasts across different models^26^. Note that OBH feedback has not been adequately included in most coupled models used for the real-time prediction of ENSO, which can be a clear source of model biases in ENSO modeling. However, the roles of these missing feedbacks in ENSO prediction biases are not fully understood^31^. This work reveals the relationships between model biases in ENSO simulations and the ways these feedbacks are represented. The results obtained from this modeling study can be used as a guide to improve ENSO predictions by adequately and coherently representing these feedbacks in coupled ocean-atmosphere models.

The demonstrated effects of FWF and OBH on the ENSO can have important implications for ENSO variability in the global warming context^32^. For example, as indicated in these HCM-based studies, a positive feedback exists between FWF and ENSO variability over the tropical Pacific. Because global warming is accompanied with large changes to precipitation patterns^25^, the induced freshwater flux changes to the ocean are expected to also exert an influence on ENSO. The results from this study can provide guidance to understand the related ENSO modulations. In addition, previous studies have focused on how global warming affects the ENSO through changes in wind stress and heat flux^25^. The demonstrated effect here indicates that global warming can also exert an influence on the ENSO through its effects on freshwater flux and salinity. Furthermore, because precipitation and freshwater flux in the tropical Pacific are a major component of global hydrological cycles, the direct modulating effect of FWF on the ENSO identified from this research also indicates a close relation between ENSO and global hydrological cycles. Thus, there can be a direct interaction between intensifying global hydrological cycles and modulating ENSO variability under global warming. Similarly, the bio-feedback identified from this study can be used to interpret possible relationships between the likely changes in ocean biology over the tropical Pacific and the induced ENSO modulations in the global warming context^33^,^34^,^35^.

Obvious differences exist between this study and previous work^16^,^17^,^23^ as already indicated by the fact that the bio-effects on ENSO variability differ significantly among different models. For example, the bio-heating variability decreases the ENSO period in this study (an indication of a negative feedback), whereas interactively represented marine biology increases the ENSO period in previous studies^17^,^23^. Additionally, the mechanisms for the bio-effects on ENSO identified from this study are different from others. For example, the SST modulations in Timmermann and Jin^13^ are realized through a direct OBH effect within the mixed layer (i.e., a change in the R_sr_ term; Supplementary section), whereas they are realized through the ocean dynamical processes induced by ocean biology in this modeling study (i.e., a differential heating induced in the vertical acting to alter the stratification and stability in the upper ocean, leading to changes in the vertical mixing). In addition, Sweeney *et al.*^16^ demonstrated the impacts of shortwave penetration depth on large-scale ocean heat transport due to off-equatorial increases in mixed layer depths. A detailed comparison is clearly needed to understand the reasons for these intermodel differences in ENSO variability. Using a simplified coupled model, it is demonstrated that the ENSO is very sensitive to ways these various FWF and OBH feedbacks are represented in coupled system of the tropical Pacific. Thus, this paper provides an illustration of how to isolate the interrelated effects induced by interannual variations in FWF and OBH and understand ENSO variability; this approach can be applied to more comprehensive modeling studies.

Due to the dominance of the ENSO signal in the tropical Pacific, there exist coherent relationships between interannual variations in SST and the responding fields (e.g., wind and FWF in the atmosphere and H_p_ in ocean biology). Therefore, their statistical relationship can be used to construct the corresponding feedback models to simply capture interannual anomalies as a response to SST variability. In this paper, we use this kind of simplified hybrid coupled model consisting of an OGCM and a simplified representation for interannual variations in the atmosphere (e.g., wind stress and freshwater flux) and in ocean biology (e.g., H_p_). Within the hybrid modeling context (Supplementary section), mean climatology fields for wind stress, freshwater flux and H_p_ are prescribed from observations, whereas their interannual variations are determined by statistical models that are constructed from historical data. Thus, this type of HCM offers a computationally efficient tool for ENSO study. In addition, the advantages of making use of such an anomaly-coupled model enable interannually varying forcing and feedback processes to be represented individually or collectively, allowing their modulating effects on the ENSO to be clearly examined. Note that in fully coupled models (e.g., the CMIP5 models), the bulk effects of these feedback processes are lumped together, making it difficult to understand individual role played by each feedback process.

This paper is intended to illustrate ENSO modulations that are induced by forcings and feedbacks within the tropical Pacific climate system. However, the combined effects identified due to interannual variations in FWF and OBH rely on only one model simulations, which can be highly model-dependent. There are large uncertainties in representing the related feedback effects and thus possible flaws in the modeling framework used in this paper. For example, the SVD-based co-variations of satellite-derived chlorophyll concentrations with SST are used to empirically depict the bulk effect of ocean biology on the penetrative solar radiation (rather than explicitly depict interactions between ocean biology and physics); then, SVD-based empirical models constructed for H_p_ and FWF are used in the HCM-based simulations, with limited SVD modes retained. Numerical experiments are performed using empirical models with parameters representing feedback intensities that are tunable. As such, there is uncertainty/sensitivity in the represented amplitude of the bio-feedback to SST changes as indicated by the feedback strength factor **α**_Hp_. For instance, to reasonably represent the intensity of the OBH effects, **α**_Hp_ is doubled in the current modeling study (Note that when taking **α**_Hp_ = 2 in the reference HCM simulation, the standard deviations of interannual H_p_ variability are well comparable to those from satellite-based estimates and thus, the OBH effect can be reasonably represented in the HCM simulation; Supplementary section). Indeed, within the statistical modeling context of H_p_, the choice of these parameters is rather arbitrary, and the modeling results are sensitive to the related parameters used (especially **α**_Hp_). Additionally, in this simplified HCM setting, interactive clouds/interannual variations of shortwave radiation are not allowed to affect the mixed layer depth and Q_pen_. As a result, the HCM indeed has systematic bias in simulating ENSO compared with observations (Supplementary section). For instance, the spatial patterns in Fig. 2 are highly distinct from the observed features compared with those of the current CGCM models (e.g., CMIP5 models). In particular, Fig. 1 shows that the largest amplitude of the interannual SST anomalies appear in the central Pacific at approximately 180 °E, although observation shows that the strongest SST variability with typical ENSO events appear in eastern tropical Pacific. Additionally, the simulated amplitude looks larger relative to observation. Thus, the results presented in this paper are preliminary and need to be validated using more realistic and comprehensive models.

## Methods

The combined effects due to interannual variations in freshwater forcing and ocean biology-induced heating are investigated using a hybrid coupled ocean-atmosphere model (HCM; Supplementary section). The HCM consists of an OGCM and a simplified representation for the atmosphere (wind stress (τ), freshwater flux and heat flux) and for ocean biology (H_p_). The OGCM is the reduced gravity, primitive equation, sigma-coordinate model of Gent and Cane (1989)^36^, which is developed specifically for studying the coupling between the dynamics and thermodynamics of the upper tropical ocean. The vertical structure of the ocean model consists of a mixed layer and a number of layers below which are specified according to a sigma-coordinate. The mixed layer depth and the thickness of the last sigma layer are computed prognostically. Several related efforts have improved this ocean model significantly, including a hybrid mixed layer model that was embedded into the OGCM^37^, a coupling of the OGCM to an advective atmospheric mixed layer (AML) model to estimate sea surface heat fluxes^38^, and inclusion of the effect of penetrative radiation on the upper tropical ocean^12^. These process-oriented studies have significantly improved simulations of ocean circulation and thermal structure^8^,^21^. The OGCM domain covers the tropical Pacific basin from 25 °S to 25 °N and from 124 °E to 76 °W, with a horizontal resolution of 1° longitude and 0.5° latitude and with 31 layers in the vertical. Sponge layers are imposed near the model southern and northern boundaries (poleward of 20 °S/N).

To demonstrate the feedback effects induced by interannually varying FWF and OBH, four experiments are performed. A reference run, denoted as FWF_inter_-OBH_inter_, is a run in which both interannual anomalies of freshwater flux and H_p_ are diagnostically determined using their empirical submodels and the related FWF and OBH feedbacks are included in the HCM-based simulations. To isolate the effects of interannually varying FWF and OBH feedbacks on ENSO, three more HCM simulations are performed. One is denoted as FWF_clim_-OBH_inter_, in which OBH is taken to be interannually varying, whereas freshwater flux is prescribed to be its seasonal climatology (i.e., the related interannually varying FWF feedback is disabled). The second is denoted as FWF_inter_-OBH_clim_, in which FWF is taken to be interannually varying, whereas H_p_ is prescribed to be its seasonal climatology (i.e., the related interannually varying ocean biology feedback is disabled). The third run is denoted as FWF_clim_-OBH_clim_, in which both FWF and OBH are taken to be its seasonal climatology without their interannually varying effects. The model experiments are detailed in the Supplementary section.

All figures in the main text and in the supplementary are created by the authors using the Grid Analysis and Display System (GrADS) which is available at http://www.iges.org/grads/grads.html.

## Additional Information

**How to cite this article**: Zhang, R.-H. *et al.* ENSO Modulations due to Interannual Variability of Freshwater Forcing and Ocean Biology-induced Heating in the Tropical Pacific. *Sci. Rep.* **5**, 18506; doi: 10.1038/srep18506 (2015).

## Supplementary Material

Supplementary Information

## Figures and Tables

**Table 1:** The standard deviations of selected anomaly fields simulated in the four HCM-based experiments, in which the interannually varying FWF and OBH effects are represented individually or collectively (FWF_inter_-OBH_inter_, FWF_clim_-OBH_inter_, FWF_inter_-OBH_clim_ and FWF_clim_-OBH_clim_).

	*FWF*_*clim*_*-OBH*_*inter*_	*FWF*_*inter*_*-OBH*_*inter*_	*FWF*_*inter*_*-OBH*_*clim*_	*FWF*_*clim*_*-OBH*_*clim*_	*Obs.*
*Niño 4 SSS*	*0.13*	*0.19*	*0.20*	*0.16*	*0.18*
*Niño 4 SST*	*0.78*	*0.96*	*1.10*	*0.85*	*0.65*
*Niño 4 τ*_*x*_	*0.016*	*0.021*	*0.025*	*0.019*	*0.18*
*Niño 4 FWF*	*0.0*	*65.4*	*78.2*	*0.0*	*86.4*
*Niño 4 H*_*p*_	*0.80*	*1.15*	*0.0*	*0.0*	*1.14*
*Niño 3 SST*	*0.62*	*0.84*	*1.02*	*0.76*	*0.67*
*Niño1* + *2 SST*	*0.46*	*0.58*	*0.68*	*0.57*	*0.67*
*Oscillation periods*	*4.3yr*	*4.5yr*	*4.7yr*	*4.6yr*	*4.8yr*

FWF_inter_-OBH_inter_ is a reference run in which both the interannually varying FWF and OBH effects are taken into account; FWF_clim_-OBH_inter_ is a run in which the OBH is taken to be interannually varying, whereas the freshwater flux is prescribed to be its seasonal climatology (i.e., the related interannually varying FWF feedback is disabled during ENSO cycles); FWF_inter_-OBH_clim_ is a run in which the FWF is taken to be interannually varying, whereas the H_p_ is prescribed to be its seasonal climatology (i.e., the related interannually varying ocean biology feedback is disabled); FWF_clim_-OBH_clim_ is a run in which both FWF and OBH are taken to be their respective seasonal climatologies without interannually varying effects. Model simulations are calculated from model year 24 to 54. Also, the corresponding values are given for observations: SSS and SST are calculated from ARGO data from year 2005 to 2013; zonal wind stress is calculated from the NCEP/NCAR reanalysis data from year 2005 to 2013; freshwater flux (P minus E; P is from the GPCP Version-2 Analysis and E is from the OAflux) from year 1979 to 2008; H_p_ from ocean color data from year 1997 to 2008. Shown are SSS, SST, zonal wind stress (τ_x_), freshwater flux and H_p_ in the Niño 4 region, SST in the Niño3 and Niño1 + 2 regions, and oscillation periods. The units are psu for SSS, °C for SST, N m^−2^ for τ_x_, mm month^−1^ for freshwater flux, and meter for H_p_.
