# Supplementary material for: ENSO Modulations due to Interannual Variability of Freshwater Forcing and Ocean Biology-induced Heating in the Tropical Pacific
Source: Sci Rep. 2015 Dec 18;5:18506. doi: 10.1038/srep18506 (PMC4683514; doi:10.1038/srep18506)
Supplement: Supplementary Information [file srep18506-s1.pdf]

# Supplementary Material of

# "ENSO Modulations due to Interannual Variability of Freshwater Forcing and Ocean Biology-induced Heating in the Tropical Pacific" by

Rong-Hua Zhang, Chuan Gao, Xianbiao Kang, Hai Zhi, Zhanggui Wang & Licheng Feng

## S1. Satellite data used

To quantify the relationships between some related interannual anomaly fields, satellite-based data are used for feedback analyses and modeling studies. Monthly precipitation (P) data are from the Global Precipitation and Climatology Project (GPCP; Adler et al., 2003); monthly evaporation (E) fields are obtained from the Objectively Analyzed Air-Sea Fluxes (OAFlux; Yu and Weller, 2007). The P and E data are used to derive freshwater flux fields over the period 1979-2008 (defined as P minus E ( P-E); the (P-E) field is positive when there is a flux from the atmosphere to the ocean).

In addition, chlorophyll (Chl) concentration data are available from the Sea-viewing Wide Field-of-view Sensor (SeaWiFS) from 1997 onward (e.g., McClain et al. 1998) and are used to represent ocean biology-induced effects on the vertical penetration of sunlight in the upper ocean. Mathematically, the attenuation of incoming solar radiation with depth follows an exponential decline in the upper ocean. Below the ocean surface, the absorption and penetration of solar radiation are wavelength ($\lambda$)-dependent, which is attributed to pure water and biogenic effects. Correspondingly, the related absorption coefficients can be expressed as $K_w(\lambda)$ and $K_{Bio}(\lambda)$, where $\lambda$ is the wavelength. As in Murtugudde et al. (2002), a single absorption coefficient in the ocean is used to account for the

average attenuation over the visible band (380-700 nm), written as $Q_{sr}(z) = \gamma \cdot \exp(-K_p \cdot z)$, where $\gamma$ is the fraction of the radiation available to vertically penetrate to depths beyond the first centimeters below the sea surface. The inverse of the attenuation coefficient $K_p$ is defined as $H_p$ ($Q_{sr}(z) = \gamma \cdot \exp(-z / H_p)$), representing a penetration depth of solar radiation in the upper ocean. Following Murtugudde et al. (2002) and Ballabrera-Poy et al. (2007), $H_p$ can be calculated from Chl concentrations using the following empirical relationship: $K_p(x, y) = K_w + a \cdot Chl(x, y)^b$ , with $\gamma = 0.33$, $K_w = 0.027$ m$^{-1}$, $a = 0.0518$ m$^{-1}$ /(mg m$^{-3}$)$^b$, and $b = 0.428$. The estimated $H_p$ field exhibits coherent variations associated with the ENSO. Thus, these data are used to characterize interannual $H_p$ variability in the tropical Pacific and to quantify its relationships with SST fields (Zhang et al. 2009, 2011). Furthermore, these data can be used to construct parameterization schemes to represent the related feedbacks.

## S2. An empirical model for interannual freshwater flux variability

Interannual freshwater flux anomalies over the tropical Pacific are dominated by ENSO signals. As indicated in Fig. s1, interannual variations in freshwater flux closely follow those in sea surface temperature (SST) during ENSO evolution. During El Niño, a warm SST anomaly is related to an increase in P (a positive freshwater flux anomaly into the ocean). During La Niña, a cold SST anomaly is accompanied with a negative freshwater flux anomaly (a net loss of freshwater from the ocean), which is primarily attributed to a deficit in P. Thus, a coherent pattern exists between interannual variations in SST and freshwater flux over the tropical Pacific, with a dominant SST control on freshwater flux.

The satellite-based P and E data are used to empirically parameterize interannual FWF variability in the tropical Pacific (Zhang and Busalacchi 2009). We adopt a statistical method to construct a

feedback model for interannual freshwater flux variability ( $(P\text{-}E)_{inter}$) as a response to ENSO-induced SST anomalies. To determine optimized empirical modes of their co-variability, a singular value decomposition (SVD) analysis is performed using historical SST and freshwater flux anomaly fields during the period 1979-2008 (a total of 30 years). Then, an empirical model for $(P\text{-}E)_{inter}$ can be developed using the derived spatial eigenvectors of the SVD modes (e.g., Zhang et al. 2006). The seasonality is taken into account by constructing seasonally dependent models for $(P\text{-}E)_{inter}$: the SVD analyses are performed separately for each calendar month, and thus the $(P\text{-}E)_{inter}$ model consists of 12 different sub-models, one for each calendar month (e.g., Zhang et al. 2003 and Zhang and Zebiak 2004). In the consideration of the sequence of the singular values and the reconstruction testing of the freshwater flux anomaly fields from SST anomalies, the first five leading SVD modes are retained for the empirical model to have reasonable amplitude in estimating $(P\text{-}E)_{inter}$ fields. Thus, given a SST anomaly, interannual freshwater flux anomalies can be empirically determined in association with ENSO.

## S3. An empirical model for the penetration depth ($H_p$) to represent ocean biology-induced heating effects

Recent modeling studies have demonstrated that ocean biology can potentially affect the climate through its effect on the vertical penetration of solar radiation in the upper ocean; the penetration depth ($H_p$) has been introduced to quantify the effects, a primary parameter in coupling biology to physics in the ocean (e.g., Zhang et al. 2009). Following Murtugudde et al. (2002) and Ballabrera-Poy et al. (2007), $H_p$ can be derived using chlorophyll content data that are available from ocean color imagery from 1997 onward (e.g., McClain et al. 1998). The total $H_p$ field is separated into its climatological part ( $\overline{H_p}$ ) and interannual anomaly part ( $H'_p$ ), written as $H_p = \overline{H}_p + H'_p$ . An example for an La Niña condition is shown in Fig. s2 for fields of chlorophyll

(Chl) concentration from the SeaWiFS and of the derived $H_p$. Coherent relationships exist between interannual variations in $H_p$ and SST during ENSO cycles.

Furthermore, based on the fact that interannual $H_p$ anomalies in the tropical Pacific are dominated by ENSO signal and exhibit a coherent co-variability pattern with SST during ENSO cycles, an empirical model for interannual $H_p$ variability was constructed empirically from a SVD analysis (Zhang et al. 2011), written as $H'_p = \alpha_{Hp} \cdot F_{Hp}(SST_{inter})$, in which $F_{Hp}$ represents the SVD-based statistical relationships between interannual variations in $H_p$ and SST, and $\alpha_{Hp}$ is a rescaling parameter that is introduced to represent the amplitude of interannual $H_p$ variability.

Note that interannual $H_p$ anomalies calculated from a given SST anomaly are somewhat weaker compared with those observed when $\alpha_{Hp} = 1.0$ is taken (e.g., Zhang et al. 2011). In the SVD analysis for the statistical $H_p$ modeling, the first five SVD modes contain only approximately 65% of the interannual $H_p$ variance. Thus, the value of $\alpha_{Hp}$ needs to be larger than 1.0 in order to compensate for the loss of the covariance and to recover the strength of interannual $H_p$ variability in the SVD-based empirical modeling. When taking $\alpha_{Hp}=2$ in the reference HCM simulation, the standard deviations of interannual $H_p$ variability are 1.15 m and 0.49 m in the Niño 4 and Niño 3 regions, respectively, which are comparable to those from satellite-based estimates (which are 1.14 m and 0.76 m, respectively). Thus, the OBH effect can be reasonably represented in the HCM simulation when taking $\alpha_{Hp}=2$.

Difficulties and large uncertainties exist in representing bio-effects in climate models. For example, interannual $H_p$ variations and the related bio-climate feedback effects have not been adequately represented in coupled ocean-atmosphere models. Thus, the empirical $H_p$ model reconstructed from chlorophyll (Chl) concentration data can be used to characterize interannual $H_p$ variability in the tropical Pacific. As such, interannual variations in $H_p$ can be diagnostically depicted from those in SST to represent bio-climate feedbacks in the tropical Pacific. The statistical modeling approach allows the determination of interannual $H_p$ variability without explicitly involving a marine

ecosystem model; the related ocean biology-induced heating effect can be taken into account for the coupled ocean-atmosphere modeling of the tropical Pacific.

## S4. A hybrid coupled model (HCM)

To demonstrate the combined effects of the two feedbacks, an HCM is used consisting of an ocean general circulation model (OGCM) and an empirical model for interannual wind stress anomalies (details of the HCM can be found in Zhang et al. (2006, 2009) and Zhang and Busalacchi (2009)). As shown in Fig. s3, the related empirical submodels for the interannual variability of FWF and $H_p$ are included to represent FWF and OBH-related climate feedbacks (Zhang and Busalacchi 2009; Zhang et al. 2009, 2011). Additionally, the related interannually varying feedbacks can be alternatively turned on or off in the HCM simulations, allowing their effects to be examined individually and collectively.

Within the context of an HCM, the total wind stress forcing to the ocean can be separated into its climatological part ($\tau_{clim}$) and interannual anomaly part ($\tau_{inter}$), written as: $\tau=\tau_{clim}+\alpha_\tau \bullet \tau_{inter}$. Similarly, the total freshwater exchange between the atmosphere and ocean (P minus E) is separated into its climatological part and interannual anomaly part: (P-E) = $(P\text{-}E)_{clim}+\alpha_{FWF} \bullet (P\text{-}E)_{inter}$; also, $H_p$ is written as $H_p = \overline{H_p} + \alpha_{Hp} \bullet H_p'$. Climatological parts ($\boldsymbol{\tau}_{\mathbf{clim}}$, $(P\text{-}E)_{clim}$ and $\overline{H_p}$ ) are all prescribed using their long-term seasonally varying fields from observations; interannual anomalies ($\tau_{inter}$, $(P\text{-}E)_{inter}$, and $H'_p$) are calculated using their corresponding empirical submodels derived from historical data. Some scalar parameters ($\boldsymbol{\alpha}_{\boldsymbol{\tau}}$, $\boldsymbol{\alpha}_{FWF}$ and $\boldsymbol{\alpha}_{Hp}$) are introduced to represent the related feedback strength (see details in Zhang et al. (2006, 2009) and Zhang and Busalacchi (2009)). For example, taking $\boldsymbol{\alpha}_{\boldsymbol{\tau}}$=1.2, $\boldsymbol{\alpha}_{FWF}$=1.0 and $\boldsymbol{\alpha}_{Hp}$=2.0 leads to HCM simulations that have reasonable interannual variations with adequately represented feedback intensity.

The OGCM, initiated from the World Ocean Atlas (WOA01) temperature and salinity fields, is integrated for 20 years using atmospheric climatology forcing fields. The coupled ocean-atmosphere

experiment is then started from this long-term OGCM spinup run, with an imposed westerly wind anomaly for eight months (Zhang et al. 2006). Thereafter, the evolution of the coupled system is determined solely by coupled ocean-atmosphere interactions in the system, and the coupled model is integrated for 30 years, arbitrarily denoted as model year 24. As shown in Zhang et al. (2006), the model can depict interannual oscillations associated with ENSO. The related FWF and OBH experiments, started from the end of the 30-year coupled run, are performed from model year 24 to year 54.

In this study, focus is placed on two feedbacks associated with interannual variations in FWF and OBH, which can be represented individually and/or collectively in the HCM. In particular, the related interannually varying feedback effects parameterized from satellite data allow for a non-local, SST-dependent, and spatially and interannually varying depictions in the tropical Pacific. For example, the statistical $H_p$ model is an SST-dependent model that represents a feedback from ocean biology to the climate system as well as an active bio-climate interaction during ENSO cycles.

## S5. The ocean-biology-induced heating terms

The effects of interannual $H_p$ variability on the vertical penetration of solar heat flux in the upper ocean can be quantified as follows. In the formulation of the OGCM (e.g., Chen et al. 1994), several ocean biology-related heating terms in the upper ocean are explicitly associated with $H_p$, including the penetrative solar radiation flux throughout the bottom of the mixed layer ($Q_{pen}$) and the absorbed part within the mixed layer ($Q_{abs}$), which can be written as

$$Q_{pen}(H_m, H_p) = Q_{sr}[\gamma \exp(-H_m / H_p)]$$

$$Q_{abs}(H_m, H_p) = Q_{sr}[1 - \gamma \exp(-H_m / H_p)]$$

where $Q_{sr}$ is the incoming solar radiation flux at the sea surface, $H_m$ is the mixed layer depth, $H_p$ is the penetration depth in the vertical, and $\gamma$ is a constant (=0.33) denoting the fraction of the radiation

available to penetrate to depths beyond the first few centimeters of the sea surface. The differences between $Q_{sr}$ and $Q_{pen}$ determine the temporal rate of change of the mixed layer temperature ($R_{sr}$), which is written as

$$R_{sr}(H_m, H_p) = Q_{sr}[1 - \gamma \exp(-H_m / H_p)] / (\rho_0 c_p H_m)$$

where $C_p$ is the heat capacity, and $\rho_0$ is the density of sea water. As explicitly expressed here, these heating terms are functions of $H_p$ and $H_m$. For example, $Q_{pen}$ exponentially decreases with $H_m$ but increases with $H_p$. Thus, the structure and variation of $H_m$ and $H_p$ exert a direct influence on $Q_{pen}$: a decrease (increase) in $H_m$ leads to a corresponding increase (decrease) in $Q_{pen}$; by contrast, a decrease (increase) in $H_p$ acts to reduce (increase) the value of $Q_{pen}$ (having an effect on $Q_{pen}$ that is opposite to $H_m$). Note that these heating terms are related to each other and are not independent. For instance, $Q_{abs}$ is the net difference between $Q_{sr}$ and $Q_{pen}$; correspondingly, an increase in $Q_{pen}$ is accompanied with a decrease in $Q_{abs}$; also, $R_{sr}$ is proportional to $Q_{abs}$ and is additionally modulated by $H_m$ (i. e., aside from the exponential dependence on $H_m$ and $H_p$, $R_{sr}$ is inversely proportional to $H_m$ because $H_m$ appears additionally in the denominator). Additionally, note that in our HCM-based simulations, $Q_{sr}$ is prescribed as a seasonally varying climatological field without interannual variability; thus, any interannual variation in these heating terms is attributed to that in $H_m$ and $H_p$.

The relationships of interannual $H_p$ variability with these related heating terms simulated in $FWF_{inter}$-$OBH_{inter}$ are used to illustrate its direct effects on the biology-mediated solar radiation uptake in the upper ocean. Fig. s4a presents interannual $Q_{pen}$ anomalies along the equator from $FWF_{inter}$-$OBH_{inter}$. As seen, $Q_{pen}$ undergoes interannual fluctuations during ENSO cycles, characterized by a see-saw pattern zonally along the equator, with a zero crossing line at approximately 150 °W (also see Fig. 3c). This is primarily attributed to interannual $H_m$ anomalies (Fig. 3b) that exhibit a similar seesaw pattern along the equator as seen from a comprehensive composite analysis. In addition, Figs.

s5-6 show the spatial patterns of one particular El Niño episode in December of model year 31 and La Niña episode in December of model year 33, respectively. During La Niña, for example, a negative $Q_{pen}$ anomaly (Fig. s6f) is seen in the western-central equatorial Pacific where the mixed layer is anomalously deep (Fig. s6e; a low $Q_{pen}$ value indicating less vertical penetration of solar radiation throughout the bottom of the mixed layer). During El Niño, a positive $Q_{pen}$ anomaly (Fig. s5f) emerges in the western-central equatorial Pacific where the ML becomes anomalously shallow (Fig. s5e; a high $Q_{pen}$ value indicating more penetration throughout the bottom of the mixed layer and thus more heating directly to the subsurface layers). An opposite anomaly pattern is seen in the eastern equatorial region (east of 150 °W), where interannual $Q_{pen}$ variability is characterized by a positive anomaly during La Niña and a negative anomaly during El Niño. As clearly evident, interannual variations in $Q_{pen}$ are negatively correlated with those in $H_m$ during ENSO evolution.

Although the interannual variability of $Q_{pen}$ is dominated by that of $H_m$, $H_p$ can play a significantly modulating role in $Q_{pen}$, which is mostly seen in the western-central basin (Fig. s4). In this region, the amplitude of interannual variability of $H_p$ can be approximately 10-20% as large as that of $H_m$ (Fig. 1; Zhang and Busalacchi 2009). Furthermore, the interannual variations in $H_m$ and $H_p$ tend to be out of phase in the region (Figs. 3a and 3b), leading to effects on $Q_{pen}$ that are in phase (Fig. 3c).

To more clearly illustrate the interannual $H_p$ effect on $Q_{pen}$, $Q_{pen}$ can be diagnostically calculated in two ways using outputs from the HCM simulation, one with the prescribed climatological $H_p$ field being used, denoted as $Q_{pen}(H_m, \overline{H}_p)$, and another with the interannually varying $H_p$ field being used, denoted as $Q_{pen}(H_m, H_p)$. Fig. s4 displays a comparison for the calculated $Q_{pen}$ fields along the equator. By looking at the differences between $Q_{pen}(H_m, H_p)$ and $Q_{pen}(H_m, \overline{H}_p)$ in Fig. s4c, it is evident that the interannual $Q_{pen}$ variability is significantly enhanced by the effect of the interannual $H_p$ variability in the western-central equatorial region. For instance, during La Niña (Fig. s6), the $H_m$ anomaly is positive in the western-central region (Fig. s6e), accompanied with a negative $Q_{pen}$

anomaly (Fig. s6f). At this time, a negative $H_p$ anomaly is seen in the western-central basin (Fig. s6d), acting to enhance the negative $Q_{pen}$ anomaly (Fig. s6f). During El Niño (Fig. s5), an opposite pattern is seen among these anomaly fields. As a result, interannual $H_p$ anomalies tend to enhance $Q_{pen}$ variability in the western-central Pacific during ENSO cycles, making it more positive during El Niño (Fig. s5f) and more negative during La Niña (Fig. s6f).

A quantitative estimate can be made based on the HCM simulation. For example, the standard deviation (std) of interannual $H_p$ variability averaged between 0.5°N-0.5°S and 160°E-160°W is 1.59 m in $FWF_{inter}$-$OBH_{inter}$. Correspondingly, the std of interannual $Q_{pen}$ variability calculated in the same region is 2.50 W $m^{-2}$ for a case in which the interannual $H_p$ effect is not included (i.e., $H_p$ is specified as a seasonally varying field in the calculation of $Q_{pen}$, as shown in Fig. s4b), and it becomes 4.01 W $m^{-2}$ for a case in which the interannual $H_p$ effect is explicitly taken into account (Fig. s4a). Thus, the interannual variability of $Q_{pen}$ is increased by approximately 60% due to the contribution from that of $H_p$. Evidently, interannual $H_p$ variability is seen to make a significant difference in determining interannual variations in $Q_{pen}$ over the western-central equatorial Pacific.

In the eastern basin (east of 150°W), the amplitude of interannual variations in $H_p$ is much smaller relative to that in $H_m$, the interannual $Q_{pen}$ variability is dominated by the $H_m$ effect there, with a small offset by $H_p$. During El Niño when the ML is deep in the eastern basin (Fig. s5e), the $Q_{pen}$ anomaly tends to be negative (Fig. s5f; an indication of less penetration throughout the bottom of the mixed layer and thus less direct heating in the subsurface layers); the effect of the positive $H_p$ anomaly (Fig. s5d) leads to a slightly reduced negative $Q_{pen}$ anomaly (thus being less negative; Fig. s5f). As a result, interannual $Q_{pen}$ variability is reduced slightly by the $H_p$ effect in the eastern equatorial Pacific (generally less than 10% as shown in Fig. s5).

## S6. Interannual anomaly patterns during individual El Niño and La Niña events

Spatial patterns of some related interannual anomaly fields are shown in Figs. s5-s6 for one particular El Niño and La Niña conditions simulated in $FWF_{inter}$-$OBH_{inter}$, which can be compared with those in Fig. 2 obtained from the composite analysis. Large interannual anomalies of freshwater flux and $H_p$ are seen across the tropical Pacific basin, with their coherent space-time relationships with SST.

The El Niño conditions (Fig. s5) are characterized by a warm SST anomaly in the central and eastern equatorial Pacific (Fig. s5a), accompanied by a positive freshwater flux anomaly (Fig. s5b; an anomalous flux into the ocean), a negative SSS anomaly (a freshening; Fig. s5c) and a shallow mixed layer (Fig. s5e) in the western-central regions, respectively. The freshwater flux anomaly tends to modulate these El Niño-produced anomaly patterns. That is, the positive FWF anomaly (Fig. s5b) has direct effects on SSS (Fig. s5c), acting to freshen the mixed layer in the western-central basin, which stabilizes the upper layer and reduces the strength of the vertical mixing and entrainment of subsurface waters into the mixed layer. These positive freshwater flux anomaly-induced oceanic processes tend to warm SSTs in the western-central basin (Fig. s5a), leading to more warming during El Niño. Thus, the relationships between changes in SST and FWF indicate a positive feedback on interannual time scales.

Additionally, during El Niño, the reduction of ocean biology activity/productivity gives rise to a positive $H_p$ anomaly in the western-central and eastern equatorial Pacific, which is accompanied by a negative anomaly in the far western regions (Fig. s5d). Accordingly, the positive $H_p$ anomaly during El Niño leads to an increased vertical penetration of sunlight throughout the bottom of the ML (a positive $Q_{pen}$ anomaly); the positive interannual $H_p$ anomaly (Fig. s5d) acts to make $Q_{pen}$ more positive (Fig. s5f) during El Niño, with more heating in the subsurface layer but less heating within the ML. The OBH effect leads to a decrease in the vertical temperature contrast, which acts to destabilize the stratification, leading to an increase in the vertical mixing and making El Niño weaker. Thus, the relationships between changes in SST and OBH indicate a negative feedback on interannual time scales. Therefore,

the effects induced by the positive feedback associated with FWF and the negative feedback associated with OBH tend to counteract each other.

## S7. Model validations

The roles of the FWF and OBH-induced feedbacks in modulating ENSO are derived from the interrelationships among some related anomaly fields simulated from the HCM, and are supported by sensitivity experiments. So, the HCM performance is critically important in realistically capturing ENSO evolution as observed in nature. Some quantitative model validations have already been presented in Table 1 for the standard deviations of some interannual anomaly fields and in Fig. 5 for the oscillation period. More model validations are presented in this subsection to show that our simplified HCM is reasonably well in depicting mean ocean climatology and interannual variability in the tropical Pacific relative to observations (The Argo products during the period from 2005 to 2013 are used, which are available directly from the International Pacific Research Center/Asia-Pacific Data-Research Center (IPRC/APDRC)). Furthermore, the HCM-based simulations are compared with those from more comprehensive coupled general circulation models (CGCMs) as represented by the GFDL ESM2M (Dunne et al. 2013; The GFDL Earth System Model with Modular Ocean Model, version 4, which a CMIP5-type model; We make use of pre-industrial control (Pi-control) scenario simulations whose data are available in the Program for Climate Model Diagnosis and Inter-comparison (PCMDI) Earth System Grid at http://pcmdi3.llnl.gov/esgcet). These are also briefly examined in the subsection.

### S7.1 A comparison between interannual variations observed and simulated

Fig. s7 exhibits one more example for interannual anomaly fields of zonal wind stress, sea surface salinity and the mixed layer depth simulated from the HCM. Together with Figs. 1-2 and other plots

presented in the paper, a comprehensive space-time evolving pattern can be seen for ENSO cycles in the HCM. The corresponding observed patterns are shown in Figs. s8-s10, including the spatial patterns during El Niño event (Fig. s9) and La Niña event (Fig. s10). The spatial features simulated in the HCM are comparable well with those observed. However, it seems obvious that the HCM has bias in simulating ENSO relative to observations (Figs. s8-s10). The spatial patterns in Figs. 1-2 and Figs. s5-s6 are much distinct from the observed features. For example, Fig. 1 shows that the largest amplitude of the interannual SST anomalies simulated appear in the central Pacific around 180$^o$E, although observation shows the largest SST anomalies with typical ENSO events appear in the eastern tropical Pacific. In addition, the simulated amplitude looks larger compared with observations.

As noted above, the simplified HCM has bias and exhibits disagreements which may influence conclusions qualitatively. For example, the 1st EOF SST mode from the HCM simulation shows the central Pacific (CP) type of El Niño, whereas the observed SST shows the canonical El Niño. However, the simulated patterns of SSS and MLD looks similar to observation.

### S7.2 A composite analysis

As described in the main text, a composite analysis is performed to depict common features of the spatial pattern and their relationships for El Niño and La Niña events in the HCM simulation (Fig. 2). The general characters derived are similar to the individual case-based analyses as shown in Figs. s5-s6. Similar composite analyses are performed for observations and comprehensive coupled general circulation model (CGCM) simulations. For example, the corresponding results analyzed from ARGO and from the GFDL ESM2M (the GFDL Earth System Model, which a CMIP5-type model) are shown in Figs. s11-s12. As seen, the spatial patterns of these related anomaly fields and their relationships simulated from the HCM match well with corresponding observations and the GFDL ESM2M-based analyses. In particular, the patterns of SSS and MLD simulated from the HCM (Figs. 2c-d) look similar

to those observed. This indicates that our simplified HCM is doing reasonably well in simulating ENSO evolution relative to observations and CGCM simulations. Although the simplified coupled model actually has bias, it may capture some essential feedbacks.

**S7.3 The ENSO phase locking with seasons**

As well-known, observed interannual SST anomalies in the central-eastern equatorial Pacific exhibit a pronounced dependence on seasons, with the weakest variability in April and strongest variability in December (e.g., Latif et al. 2001). Fig. s13 illustrates the standard deviations of the Niño 3 SST anomalies simulated from the HCM as a function of the calendar months. There is an indication of seasonal variation of the ENSO-related SST variability in the HCM, with relatively weak amplitude in spring. However, the HCM is poor at simulating the observed ENSO peak in winter, a common bias with many other coupled models (e.g., Latif et al. 2001; Ham and Kug 2014). There is a clear need to improve model simulations in this regard.

**S7.4 The simulated mean ocean climatology**

The HCM can well depict the observed mean ocean climatology in the tropical Pacific. Some examples are given in Figs. s14-17 from the $FWF_{inter}$-$OBH_{inter}$ run, which can be compared with the corresponding observational fields and other model simulations (e.g., Latif et al. 2001; Hosoda et al. 2008; Ham and Kug 2014; Zhang 2015). Also, the mean ocean states are similar among the different HCM experiments with the related FWF and OBH feedbacks being included or not because their effects are of opposite signs between cold and warm phases of ENSO (Fig. 2 and Figs. s5-s6).

**S7.5 Long-term HCM runs**

Some extended integrations (more than 100 years) were performed using the HCM with the related

FWF and OBH feedbacks being included. One example is given in Figs. s18-19 for interannual SST anomaly fields and total SST fields simulated from the $FWF_{inter}$-$OBH_{inter}$ run. The longer simulations allow the HCM to have enough ENSO cycles. As evident, the HCM has a really regular, sustainable ENSO although no stochastic atmospheric forcing is included in the system. The simulated interannual variability exhibits similar features during different decades of time integrations, including space-time evolution, the relationships among anomaly fields, oscillation periods, and phase locking. Thus, the simulated ENSO properties do not change with time as represented by analyses based on the shorter time integration. For example, the spatial patterns of a composite of El Niño and La Niña events are very similar to those of a single El Niño and La Niña event as shown in Fig. 2 and Fig. s5. Also, the bias characteristics are similar during different decades of time integrations, including the overestimated SST variability in the central equatorial Pacific, and underestimated variability to the east. In addition, the longer simulations would allow a nonlinear rectification of ENSO-related feedback effects into the model mean ocean state. As these feedback effects are of opposite signs between the different phases of ENSO without obvious changes in ENSO asymmetry, the simulated mean ocean states are similar among the various HCM experiments during different periods of time integrations.

Overall, by comparing the HCM-based simulations to those from observations and CGCMs, more convincing evidence is given to show the fidelity of our HCM, including ENSO properties and mean ocean state. It indicates that the simplified HCM can be a good tool for this study even if it is not perfect. A case-based analysis and a comprehensive composite analysis are performed to demonstrate that our model results shown in Fig. 2 and Figs. s5-s6 are robust in capturing the common features for El Niño and La Niña events, with the general importance of the two feedbacks and related processes during ENSO cycles illustrated. Long-term integrations indicate that the HCM has a really regular, sustainable ENSO, with the same interannual variability and mean ocean state during different

decades of time integrations.

**S7.6 Significance tests for the changes in interannual variability**

Since our analyses for the changes in ENSO rely on one single experiment of one particular model, it is desirable to make sure that the results are statistically significant within this framework. We perform statistical significance tests for the changes in interannual variability between the different experiments. For example, some statistics are estimated for the changes in ENSO amplitude to evaluate if the changes in the ENSO amplitude between the different experiments are significant. Fig. s20 displays the statistics as represented by interannual SST variability (the stippling regions indicate where changes in ENSO amplitude between different experiments are statistically significant by applying the F-test).

Additionally, to see statistically significant differences in the dominant oscillation periods, we apply the F-test to the spectra estimates derived from different experiments. Table s1 presents some kind of confidence interval estimate made for ENSO periods simulated from model experiments and observation. Confidence limits are set using an F-distribution, with the null value estimated from the F-distribution being 3.59 year (yr) at the 95% significance level ($F_0$=3.59 yr). The F-values estimated from the $\text{FWF\_}_{\text{inter}}$-$\text{OBH\_}_{\text{inter}}$, $\text{FWF\_}_{\text{inter}}$-$\text{OBH\_}_{\text{clim}}$ and $\text{FWF\_}_{\text{clim}}$-$\text{OBH\_}_{\text{inter}}$ experiments are 4.7 yr, 4.2 yr and 3.7 yr, respectively. Since these F-distribution derived values are all larger than F0, the spectra peaks are all significant. Also, the dominant periods are all significantly different from each other because the F-tests exceed the upper 95% confidence limit over these frequency ranges. These significance tests for the interannual amplitudes and periods help understand how much we can trust a change in ENSO amplitudes and periods induced by the inclusion or exclusion of one specific feedback process and how the changes in ENSO are significant with such short model runs.

## Additional references


Adler, R. F. et al., 2003: The version-2 global precipitation and climatology project (GPCP) monthly precipitation analysis (1979–present), *J. Hydromet.*, **4**, 1147–1167.

Dunne, J. P., and Coauthors, 2013: GFDL’s ESM2 Global Coupled Climate–Carbon Earth System Models. Part II: Carbon System Formulation and Baseline Simulation Characteristics. *J. Climate*, **26**, 2247–2267.

Ham, Y.-G., and J.-S. Kug, 2014: ENSO phase-locking to the boreal winter in CMIP3 and CMIP5 models. *Climate Dyn.*, **43**, 305–318, doi:10.1007/s00382-014-2064-1.

Hosoda, S., Ohira, T., Nakamura, T., 2008: A monthly mean dataset of global oceanic temperature and salinity derived from Argo float observations. *JAMSTEC Rep. Res. Dev.*, **8**, 47–59.

Latif, M. et al., 2001: ENSIP: the El Niño simulation intercomparison project. *Clim. Dyn.*, **18**: 255–276.

McClain, C. R., M. L. Cleave, G. C. Feldman, W. W. Gregg, S. B. Hooker, N. Kuring, 1998: Science quality SeaWiFS data for global biosphere research. *Sea Technol*, **39**, 10–16.

Yu, L., R. A. Weller, 2007: Objectively analyzed air-sea heat fluxes for the global ice-free oceans (1981–2005), *Bull. Amer. Meteor. Soc.*, **88**, 527–539.

Zhang, R.-H., S. E. Zebiak, R. Kleeman, and N. Keenlyside, 2003: A new intermediate coupled model for El Nino simulation and prediction, *Geophys. Res. Lett.*, Vol. **30**, no. 19, 2012, doi:10.1029/2003GL018010.

Zhang, R.-H., and S. E. Zebiak, 2004: An embedding method for improving interannual variability simulations in a hybrid coupled model of the tropical Pacific ocean-atmosphere system. *J. Climate*, **17**, 2794-2812.

Zhang, R.-H., 2015: A hybrid coupled for the Pacific ocean-atmosphere system: Part I: Its formalism and basic performance. *Adv. Atmos. Sci.*, **32**: 301-318, doi: 10.1007/s00376-014-3266-5.

**Table s1** The estimates of confidence interval with some related statistics, which are calculated using interannual SST anomaly fields in the Niño3.4 region during years 2005-2013 for observation and during model years 24-54 for different model experiments.

| | SST (FWF_inter -OBH_clim) | SST(FWF_clim -OBH_inter) | SST(FWF_inter -OBH_inter) | OBS |
|---|---|---|---|---|
| Sample size | 372 | 372 | 372 | 372 |
| Sample mean | 0.2104 | 0.0495 | 0.1522 | 0.1103 |
| Sample standard deviation | 1.1160 | 0.6686 | 0.9158 | 0.7226 |
| The sampling mean error | 0.0579 | 0.0347 | 0.0475 | 0.0221 |
| Degree of confidence | 0.95 | 0.95 | 0.95 | 0.95 |
| Degrees of freedom | 371 | 371 | 371 | 371 |
| The double side of the t distribution quantile | 1.966378803 | 1.966378803 | 1.966378803 | 1.9663788 03 |
| Margin of error | 0.1138 | 0.0682 | 0.0934 | 0.0033 |
| The lower limit of confidence interval (yr) | 3.5 | 3.15 | 3.4 | 3.1 |
| The upper limit of the confidence interval (yr) | 6.2 | 5.6 | 5.7 | 5.6 |

# Figure captions for the Supplemental section

**Fig. s1** Examples of the spatial patterns of interannual anomalies of (a) SST, (b) precipitation (P), and (c) sea surface salinity (SSS) in Dec. 2006, representing an La Niña condition in the tropical Pacific, as revealed from satellite measurements (Adler et al. 2003) and Argo profiles (Hosoda et al. 2008). During El Niño, a warm SST anomaly in the equatorial Pacific is related to an increase in P over the central and eastern equatorial Pacific, with a positive freshwater flux anomaly into the ocean and a negative SSS anomaly. The contour interval is 0.5°C in (a), 2 mm day$^{-1}$ in (b), and 0.1 psu in (c).

**Fig. s2** An example illustrating ocean color measurements made by the NASA SeaWiFS satellite (upper panel). The ocean color data are used to derive chlorophyll (Chl) concentrations, a green pigment that helps phytoplankton convert sunlight into food in the marine ecosystem. For example, an ocean color with dark shades of blue denotes low Chl concentrations in the subtropical regions, whereas the green areas in the equatorial Pacific denote high Chl concentrations. The distribution and variation of Chl affect the vertical penetration of sunlight in the upper ocean, which can be simply represented by the penetration depth ($H_p$), a primary field to link ocean biology to ocean physics. Shown in the figure are the derived horizontal distributions of interannual anomalies for Chl concentrations (a) and for $H_p$ (b) in Aug. 1998, when a La Niña condition prevailed in the tropical Pacific. For example, there is a rise in Chl concentrations in the western-central equatorial basin during the La Niña event. As a result, the sunlight is trapped more within the mixed layer and thus penetrates less throughout the bottom of the mixed layer (less direct heating in the subsurface layers). On the other hand, during El Niño, there is a drop in Chl concentrations; correspondingly, the sunlight is able to penetrate deeper into the upper ocean. Thus, satellite measurements can be used to quantify the OBH effects, which are incorporated into coupled ocean-atmosphere models. Also shown is the schematic for the OB-induced climate feedback in the tropical Pacific during ENSO evolution: La

Niña induces large perturbations in physical and biogeochemical fields, including Chl and $H_p$, which acts to mediate solar radiation uptake in the upper ocean, which in turn affects La Niña conditions. The contour interval is 0.05 mg $m^{-3}$ for Chl in (a) and 1 m for $H_p$ in (b).

**Fig. s3** A schematic diagram illustrating a hybrid coupled model (HCM) used to demonstrate the combined effects of freshwater forcing (FWF) and ocean biology (OB)-induced heating (OBH) in the tropical Pacific ocean-atmosphere system. The HCM consists of an OGCM and a simplified atmospheric representation, whose three forcing fields to the ocean are included (i.e., wind stress (τ), and fluxes of freshwater and heat). The total wind stress (τ) is separated into its climatological part ($\tau_{clim}$) and its interannual anomaly part ($\tau_{inter}$): $\tau=\tau_{clim}+\alpha_{\tau}\bullet\tau_{inter}$. The total freshwater flux, represented by precipitation minus evaporation, (P-E), is also separated into its prescribed climatological part ( $(P\text{-}E)_{clim}$) and its interannual anomaly part ($FWF_{inter}$), written as $FWF = (P\text{-}E)_{clim} + \alpha_{FWF} \bullet FWF_{inter}$, which has direct effects on sea surface salinity (SSS) and buoyancy flux ($Q_B$). The heat flux (HF) is calculated using an advective atmospheric mixed layer (AML) model (Seager et al. 2005). In addition, the climate system is affected by ocean biology in the region, whose effects on ocean physics are simply represented by the attenuation depth of solar radiation in the upper ocean ($H_p$); similarly, the total $H_p$ field is separated into its climatological part ( $\overline{H_p}$ ) and interannual anomaly part ( $H'_p$ ), written as $H_p = \overline{H_p} + \alpha_{Hp} \bullet H_p{}'$ . Some scalar coefficients ($\alpha_{\tau}$, $\alpha_{FWF}$, and $\alpha_{Hp}$) are introduced to represent the strength of the corresponding feedbacks of interest. In this simplified coupled system, climatological fields ($SST_{clim}$, $(P\text{-}E)_{clim}$, and $\overline{H_p}$) are prescribed to be seasonally varying; interannual anomaly fields ($\boldsymbol{\tau}_{inter}$, $FWF_{inter}$ and $H'_p$) are diagnostically determined from their corresponding empirical models which are constructed using a singular value decomposition (SVD) analysis technique.

**Fig. s4** Interannual $Q_{pen}$ anomalies along the equator calculated (a) with and (b) without the effect of

the interannual $H_p$ anomalies explicitly taken into account, and (c) their differences. The contour interval is 2 W $m^{-2}$ in (a) and (b) and 1 W $m^{-2}$ in (c).

**Fig. s5** Horizontal patterns of interannual anomalies simulated from the reference run ($FWF_{inter}$-$OBH_{inter}$) for El Niño conditions as represented in December of model year 31: (a) SST, (b) freshwater flux, (c) SSS, (d) $H_p$, (e) the depth of the mixed layer ($H_m$), and (f) $Q_{pen}$. The contour interval is 0.5°C in (a), 20 mm $month^{-1}$ in (b), 0.1 psu in (c), 0.5 meters in (d), 4 meters in (e), and 2 W $m^{-2}$ in (f).

**Fig. s6** The same as in Fig. s5 but for La Niña conditions as represented in December of model year 33.

**Fig. s7** Interannual anomaly fields along the equator simulated from the reference run ($FWF_{inter}$-$OBH_{inter}$): (a) zonal wind stress, (b) SSS, and (c) the mixed layer depth ($H_m$). The contour interval is 0.1 dyn $cm^{-1}$ in (a), 0.1 psu in (b), and 4 meters in (c).

**Fig. s8** Interannual anomaly fields along the equator observed from ARGO during 2005-2013: (a) SST, (b) SSS, and (c) MLD ($H_m$). The contour interval is 0.5 °C in (a), 0.1 psu in (b), and 4 meters in (c).

**Fig. s9** Horizontal patterns of interannual anomaly fields observed from ARGO for El Niño conditions in October 2009: (a) SST, (b) SSS, and (c) MLD ($H_m$). The contour interval is 0.5°C in (a), 0.1 psu in (b), and 4 meters in (c).

**Fig. s10** The same as in Fig. s9 but for La Niña conditions in October 2010.

**Fig. s11** Spatial patterns of the first EOF SST mode and the corresponding regression patterns of some related anomaly fields with the principal component (PC) of the first EOF SST mode which are analyzed from observations: (a) SST, (b) FWF, (c) SSS, and (d) MLD ($H_m$). Here, observed temperature and salinity fields are from ARGO data during 2005-2013; freshwater flux field during period 1979-2008 is calculated from the Global Precipitation Climatology Project (GPCP)

Version-2 Analysis for precipitation (Adler et al. 2003) and OAflux for evaporation (Yu and Weller 2007), respectively. The units are °C for SST, mm $day^{-1}$ for FWF, psu for SSS and m for MLD.

**Fig. s12** The same as in Fig. s11 but from the GFDL-ESM2 simulation. We make use of pre-industrial control (Pi-control) scenario runs; data from the last 100 years are selected.

**Fig. s13** Simulated and observed standard deviations of the Niño 3 SST anomalies (°C) as a function of the calendar month. The HCM-based simulations are calculated from model year 24 to 54 and the observation is calculated from year 1971 to 2000.

**Fig. s14** Horizontal distributions of annual-mean fields simulated from the $FWF_{inter}$-$OBH_{inter}$ run for (a) SST, (b) SSS, (c) the mixed layer depth (MLD), and (d) the penetration depth ($H_p$). The contour interval is 1 °C for SST, 0.1 psu for SSS, 5 m for MLD, and 1 m for $H_p$.

**Fig. s15** Horizontal distributions of annual-mean fields simulated from the $FWF_{inter}$-$OBH_{inter}$ run for (a) sea level (SL) and (b) surface zonal current. The contour interval is 0.1 m in (a) and 10 cm $s^{-1}$ in (b).

**Fig. s16** (a) The SST seasonal cycle and (b) annual variations (relative to its annual mean) along the equator simulated from the $FWF_{inter}$-$OBH_{inter}$ run. The contour interval is 0.5°C in (a) and 0.3°C in (b).

**Fig. s17** The seasonal cycles along the equator for (a) MLD and (b) surface zonal currents simulated from the $FWF_{inter}$-$OBH_{inter}$ run. The contour interval is 5 m in (a) and 10 cm $s^{-1}$ in (b).

**Fig. s18** Interannual SST anomalies along the equator simulated from the $FWF_{inter}$-$OBH_{inter}$ run. The HCM was integrated for more than 100 years and the plotting is shown for model year 45 to 104. The contour interval is 0.5 °C.

**Fig. s19** The same as in Fig. s18 but for the total SST fields. The contour interval is 1 °C.

**Fig. s20** Horizontal distributions of the standard deviations for interannual SST variabilities calculated from different HCM experiments: (a) $FWF_{inter}$-$OBH_{inter}$; (b) $FWF_{inter}$-$OBH_{clim}$; (c) $FWF_{clim}$-$OBH_{inter}$. Additionally, to test if the changes in interannual SST variabilities between

$FWF_{inter}$-$OBH_{inter}$ and $FWF_{inter}$-$OBH_{clim}$, and between $FWF_{inter}$-$OBH_{inter}$ and $FWF_{clim}$-$OBH_{inter}$ are significant, we apply an F-test to these interannual SST anomaly fields. The stippling regions in (b) and (c) indicate that the standard deviations in the $FWF_{inter}$-$OBH_{clim}$ and $FWF_{clim}$-$OBH_{inter}$ simulations are significantly different from those in the $FWF_{inter}$-$OBH_{inter}$ simulation. The contour interval is 0.2 °C.

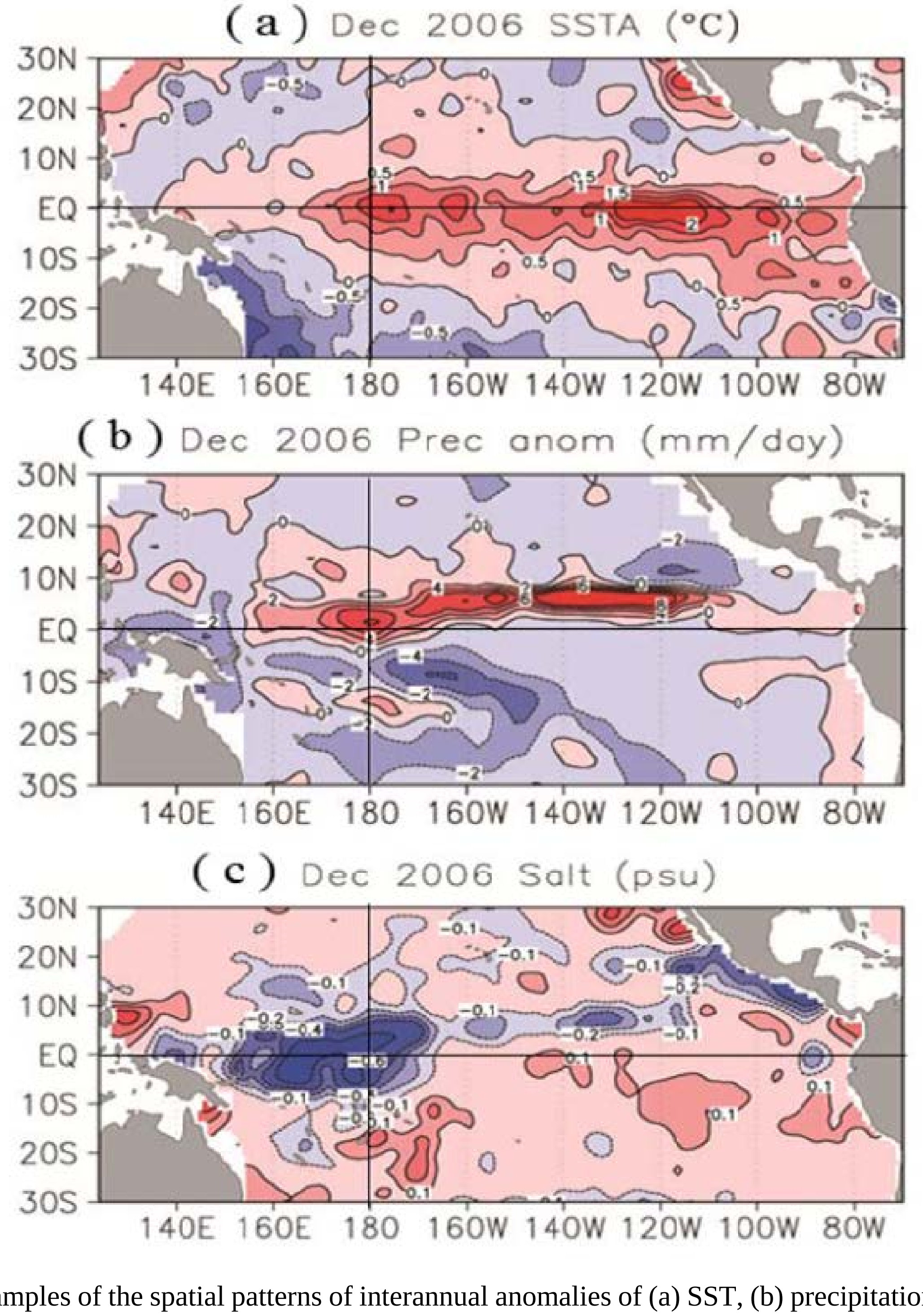


Fig. s1 Examples of the spatial patterns of interannual anomalies of (a) SST, (b) precipitation (P), and (c) sea surface salinity (SSS) in Dec. 2006, representing an La Niña condition in the tropical Pacific, as revealed from satellite measurements (Adler et al. 2003) and Argo profiles (Hosoda et al. 2008). During El Niño, a warm SST anomaly in the equatorial Pacific is related to an increase in P over the central and eastern equatorial Pacific, with a positive freshwater flux anomaly into the ocean and a negative SSS anomaly. The contour interval is 0.5°C in (a), 2 mm day$^{-1}$ in (b), and 0.1 psu in (c).

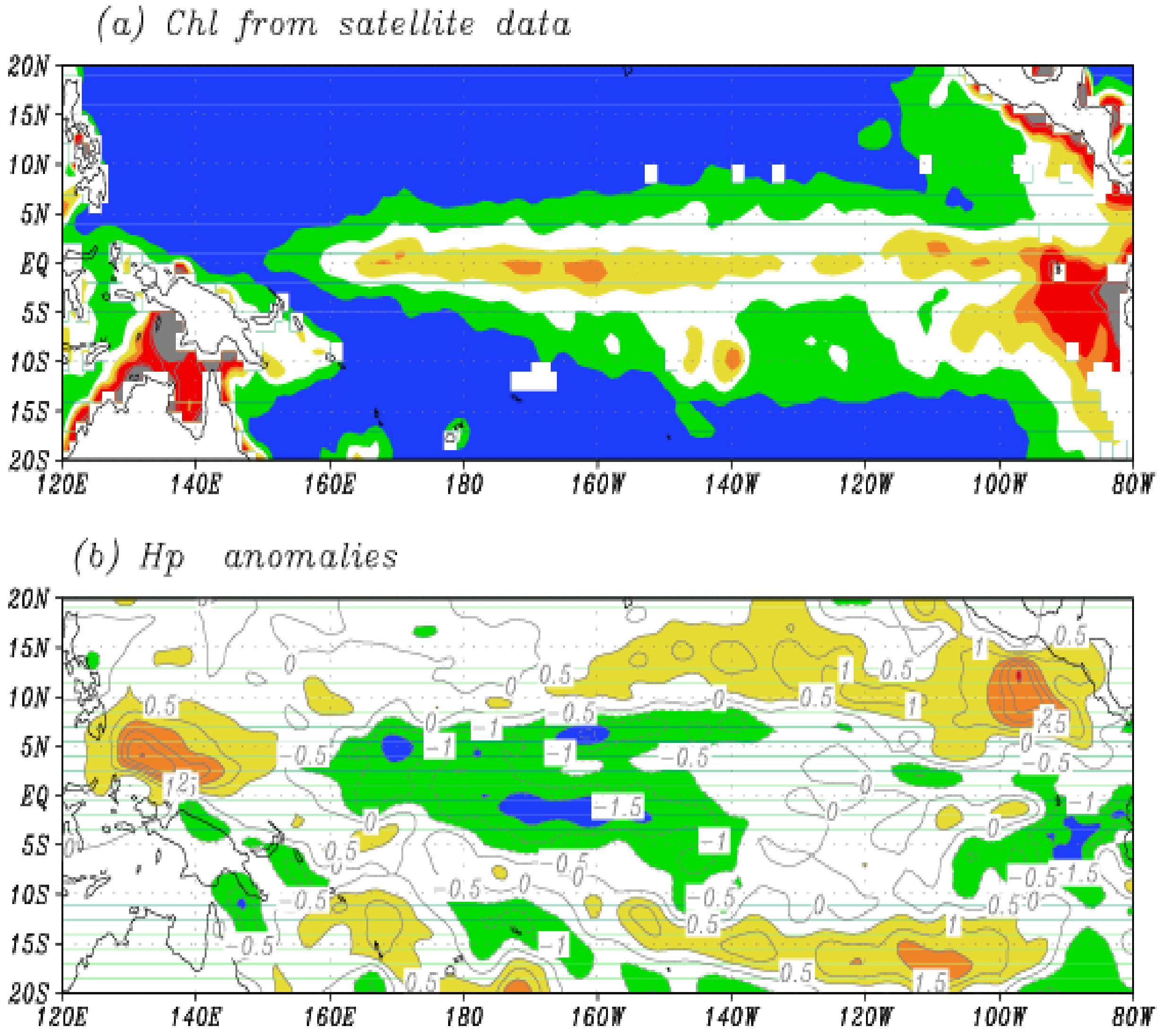


Fig. s2 An example illustrating ocean color measurements made by the NASA SeaWiFS satellite (McClain et al. 1998). The ocean color data are used to derive chlorophyll (Chl) concentrations. The distribution and variation of Chl affect the vertical penetration of sunlight in the upper ocean, which can be simply represented by the penetration depth ($H_p$), a primary field to link ocean biology to ocean physics. Shown in the figure are the derived horizontal distributions of interannual anomalies for Chl concentrations (a) and for $H_p$ (b) in Aug. 1998, when a La Niña condition prevailed in the tropical Pacific. For example, there is a rise in Chl concentrations in the western-central equatorial basin during the La Niña event. As a result, the sunlight is trapped more within the mixed layer and thus penetrates less throughout the bottom of the mixed layer (less direct heating in the subsurface layers). On the other hand, during El Niño, there is a drop in Chl concentrations; correspondingly, the sunlight is able to penetrate deeper into the upper ocean. Thus, satellite measurements can be used to quantify the OBH effects, which are incorporated into coupled ocean-atmosphere models. The contour interval is 0.05 mg $m^{-3}$ for Chl in (a) and 1 m for $H_p$ in (b).

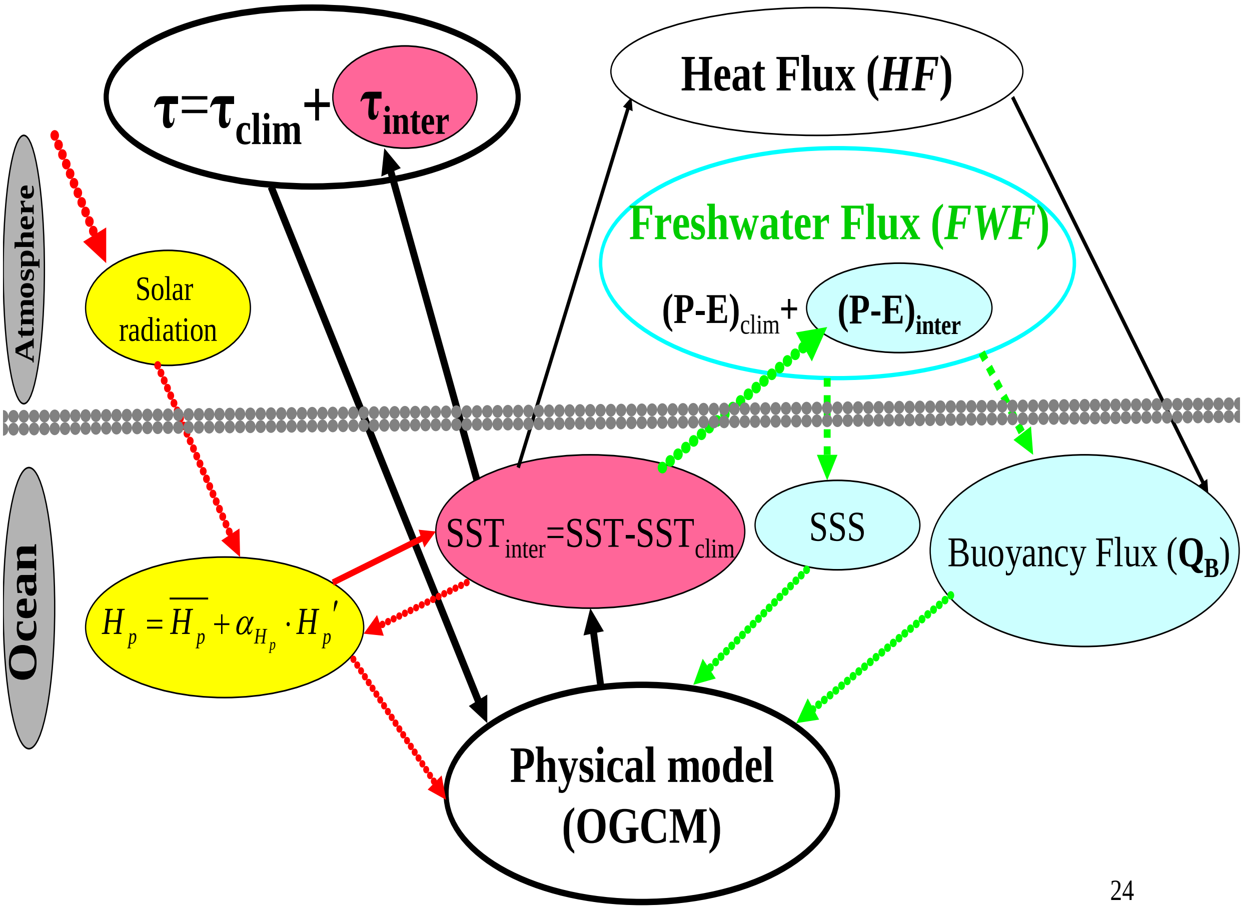


Fig. s3 A schematic diagram illustrating a hybrid coupled model (HCM) used to demonstrate the combined effects of freshwater forcing (FWF) and ocean biology (OB)-induced heating (OBH) in the tropical Pacific ocean-atmosphere system. The HCM consists of an OGCM and a simplified atmospheric representation, whose three forcing fields to the ocean are included (i.e., wind stress (τ), and fluxes of freshwater and heat). The total wind stress (τ) is separated into its climatological part ($\tau_{clim}$) and its interannual anomaly part ($\tau_{inter}$): $\tau=\tau_{clim}+\alpha_\tau \bullet \tau_{inter}$. The total freshwater flux, represented by precipitation minus evaporation, (P-E), is also separated into its prescribed climatological part ( $(P\text{-}E)_{clim}$) and its interannual anomaly part ($FWF_{inter}$), written as $FWF = (P\text{-}E)_{clim}+ \alpha_{FWF} \bullet FWF_{inter}$, which has direct effects on sea surface salinity (SSS) and buoyancy flux ($Q_B$). The heat flux (HF) is calculated using an advective atmospheric mixed layer (AML) model (Seager et al. 2005). In addition, the climate system is affected by ocean biology in the region, whose effects on ocean physics are simply represented by the attenuation depth of solar radiation in the upper ocean ($H_p$); similarly, the total $H_p$ field is separated into its climatological part ( $\overline{H_p}$ ) and interannual anomaly part ( $H'_p$ ), written as $H_p = \overline{H_p} + \alpha_{Hp} \bullet H_p'$. Some scalar coefficients ($\alpha_\tau$, $\alpha_{FWF}$, and $\alpha_{Hp}$) are introduced

to represent the strength of the corresponding feedbacks of interest. In this simplified coupled system, climatological fields ($SST_{clim}$, $(P\text{-}E)_{clim}$, and $\overline{H_p}$ ) are prescribed to be seasonally varying; interannual anomaly fields ($\boldsymbol{\tau}_{inter}$, $FWF_{inter}$ and $H'_p$) are diagnostically determined from their corresponding empirical models which are constructed using a singular value decomposition (SVD) analysis technique.

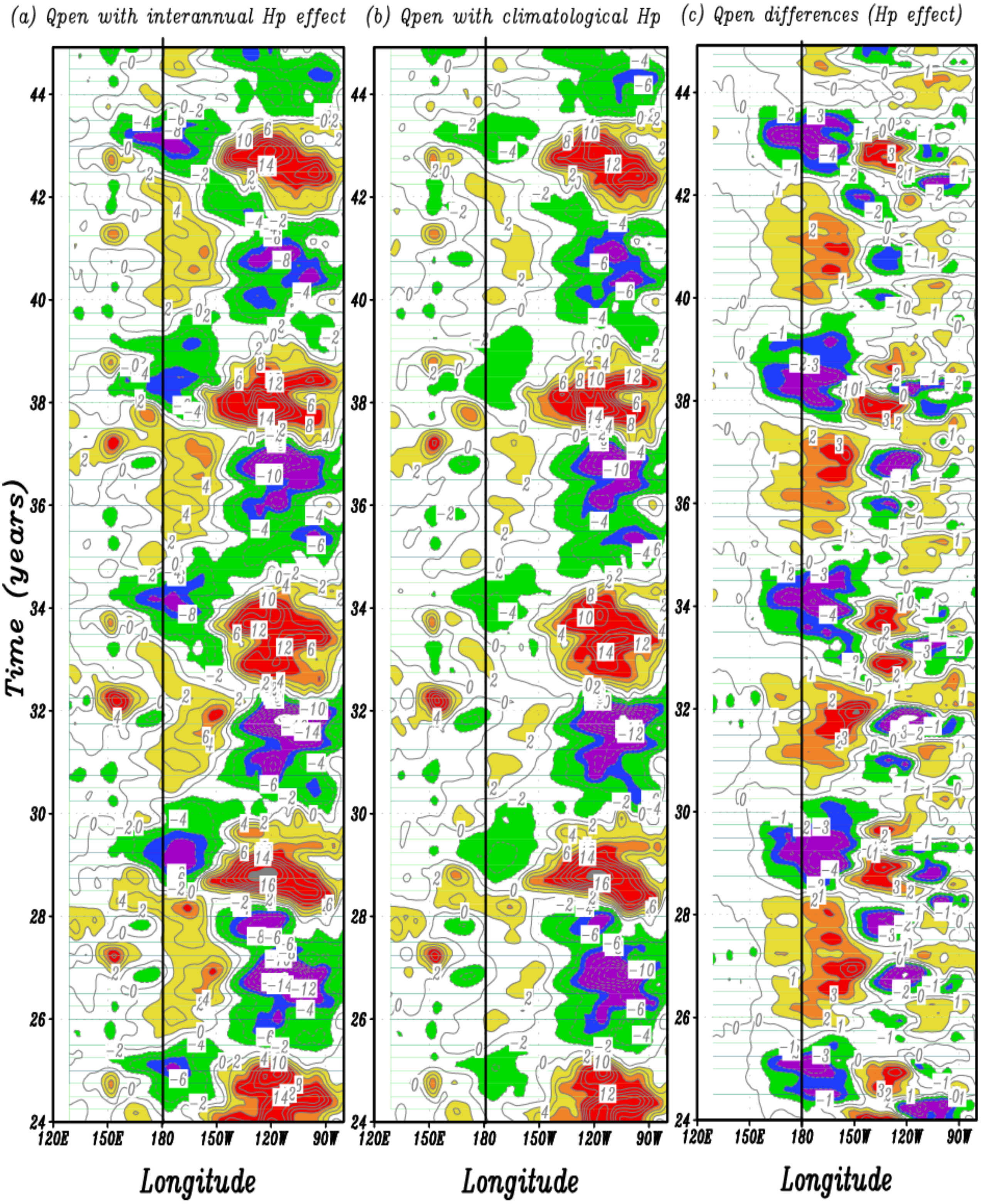


Fig. s4 Interannual $Q_{pen}$ anomalies along the equator calculated (a) with and (b) without the effect of the interannual $H_p$ anomalies explicitly taken into account, and (c) their differences. The contour interval is 2 W $m^{-2}$ in (a) and (b) and 1 W $m^{-2}$ in (c).

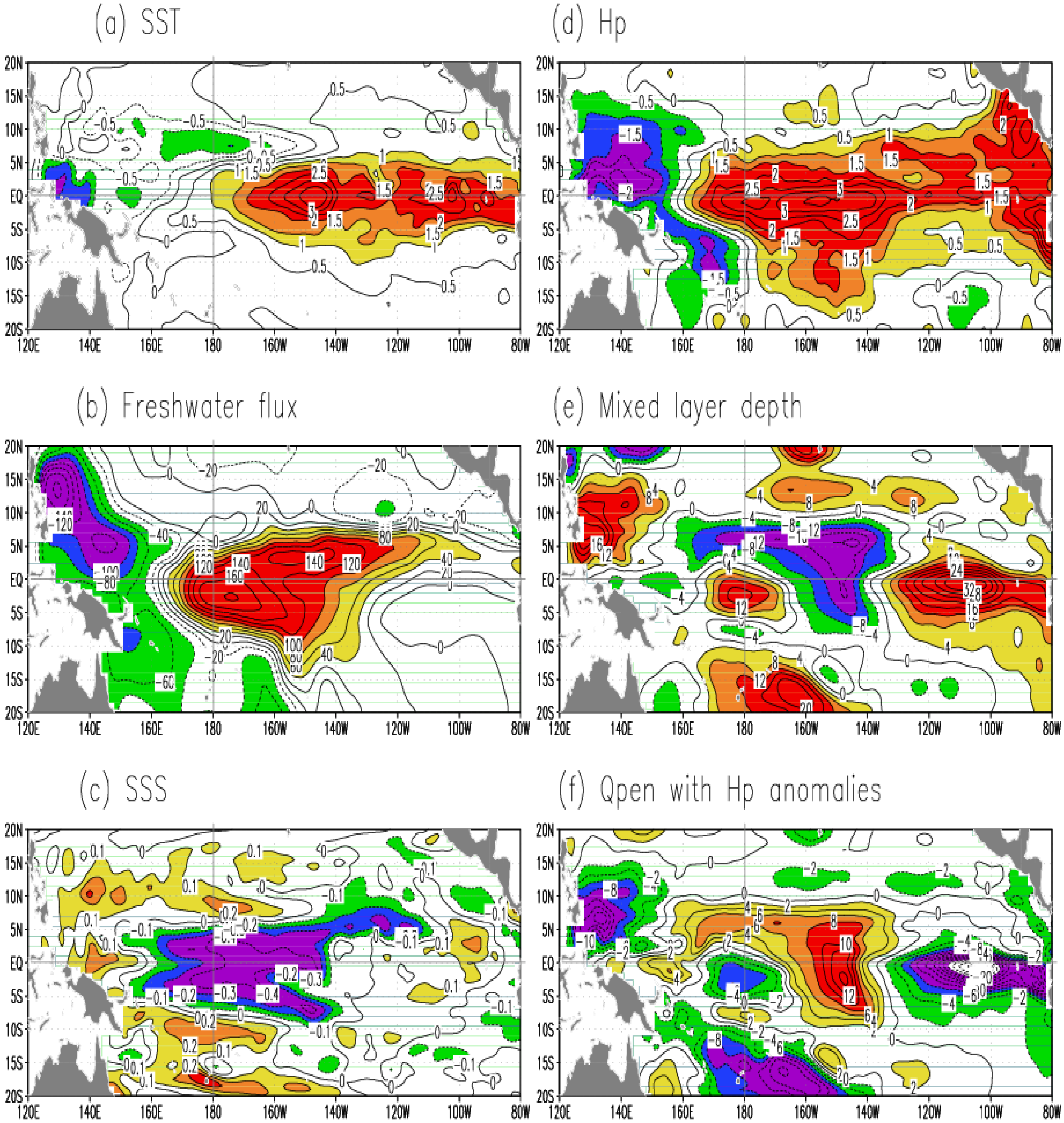


Fig. s5 Horizontal patterns of interannual anomalies simulated from the reference run ($FWF_{inter}$-$OBH_{inter}$) for El Niño conditions as represented in December of model year 31: (a) SST, (b) freshwater flux, (c) SSS, (d) $H_p$, (e) the depth of the mixed layer ($H_m$), and (f) $Q_{pen}$. The contour interval is 0.5°C in (a), 20 mm month$^{-1}$ in (b), 0.1 psu in (c), 0.5 meters in (d), 4 meters in (e), and 2 W m$^{-2}$ in (f).

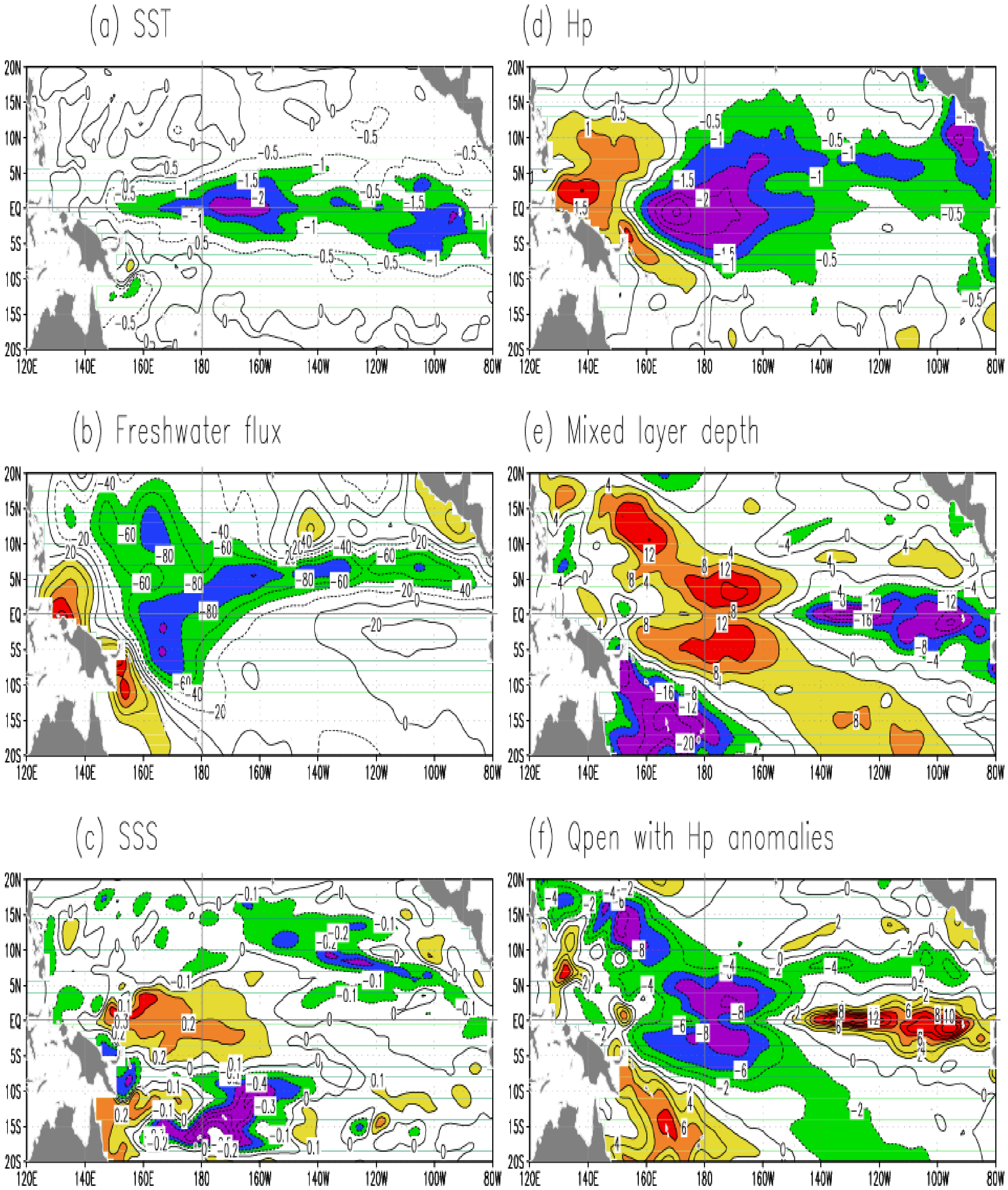


**Fig. s6** The same as in Fig. s5 but for La Niña conditions as represented in December of model year 33.

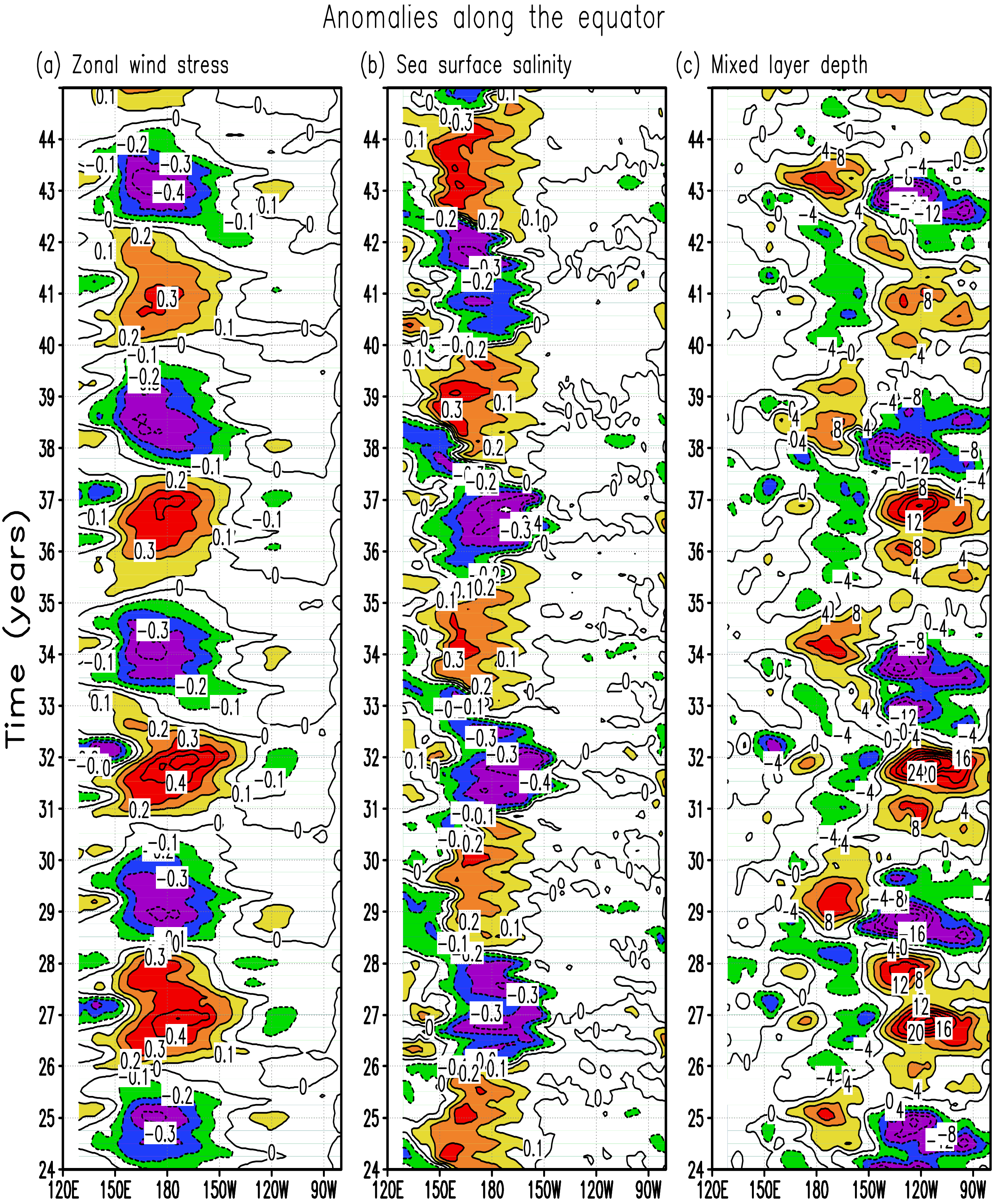


Fig. s7 Interannual anomaly fields along the equator simulated from the reference run ($FWF_{inter}$-$OBH_{inter}$): (a) zonal wind stress, (b) SSS, and (c) the mixed layer depth (MLD). The contour interval is 0.1 dyn $cm^{-1}$ in (a), 0.1 psu in (b), and 4 meters in (c).

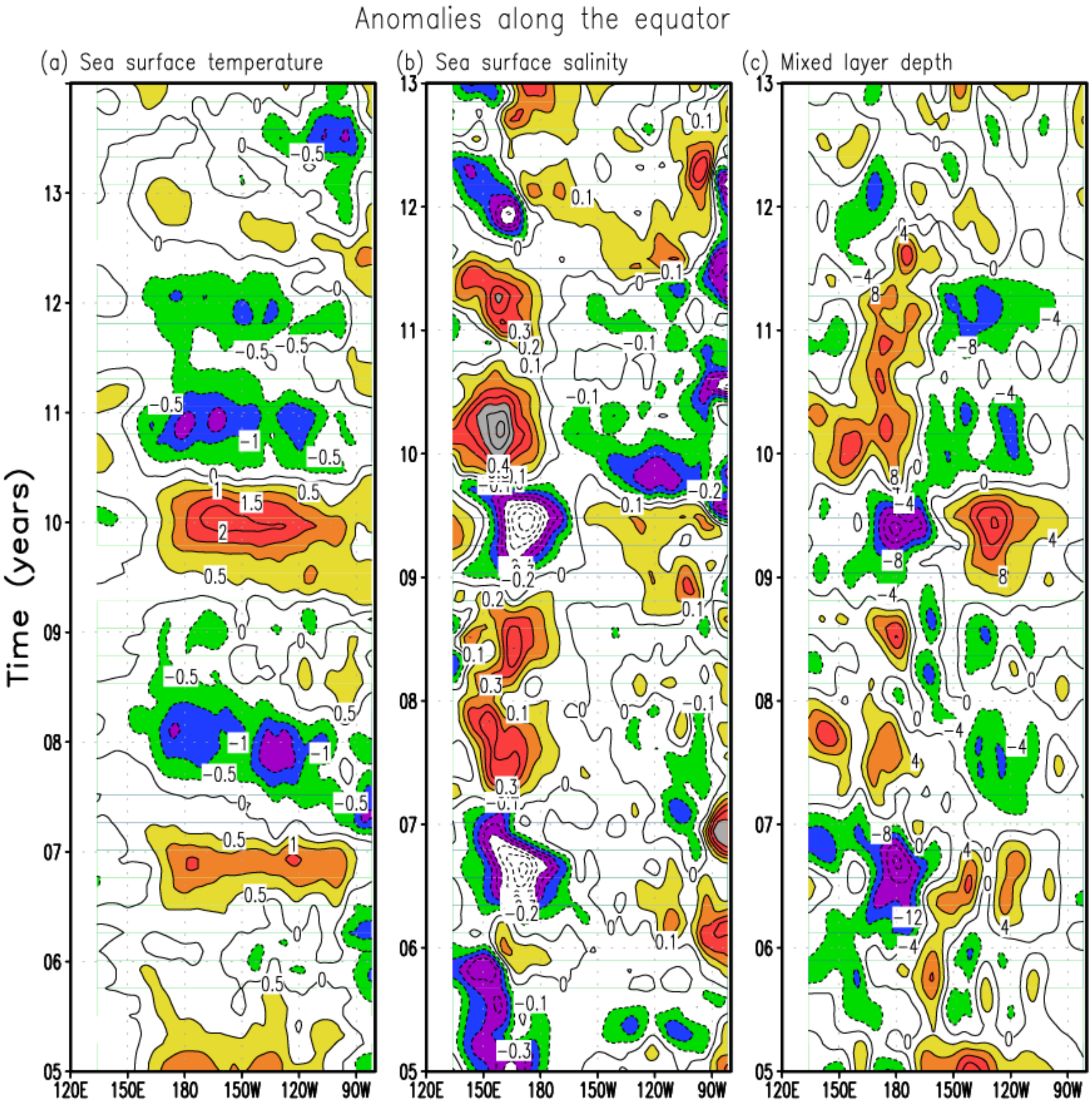


Fig. s8 Interannual anomaly fields along the equator observed from ARGO during 2005-2013: (a) SST, (b) SSS, and (c) MLD. The contour interval is 0.5 °C in (a), 0.1 psu in (b), and 4 meters in (c).

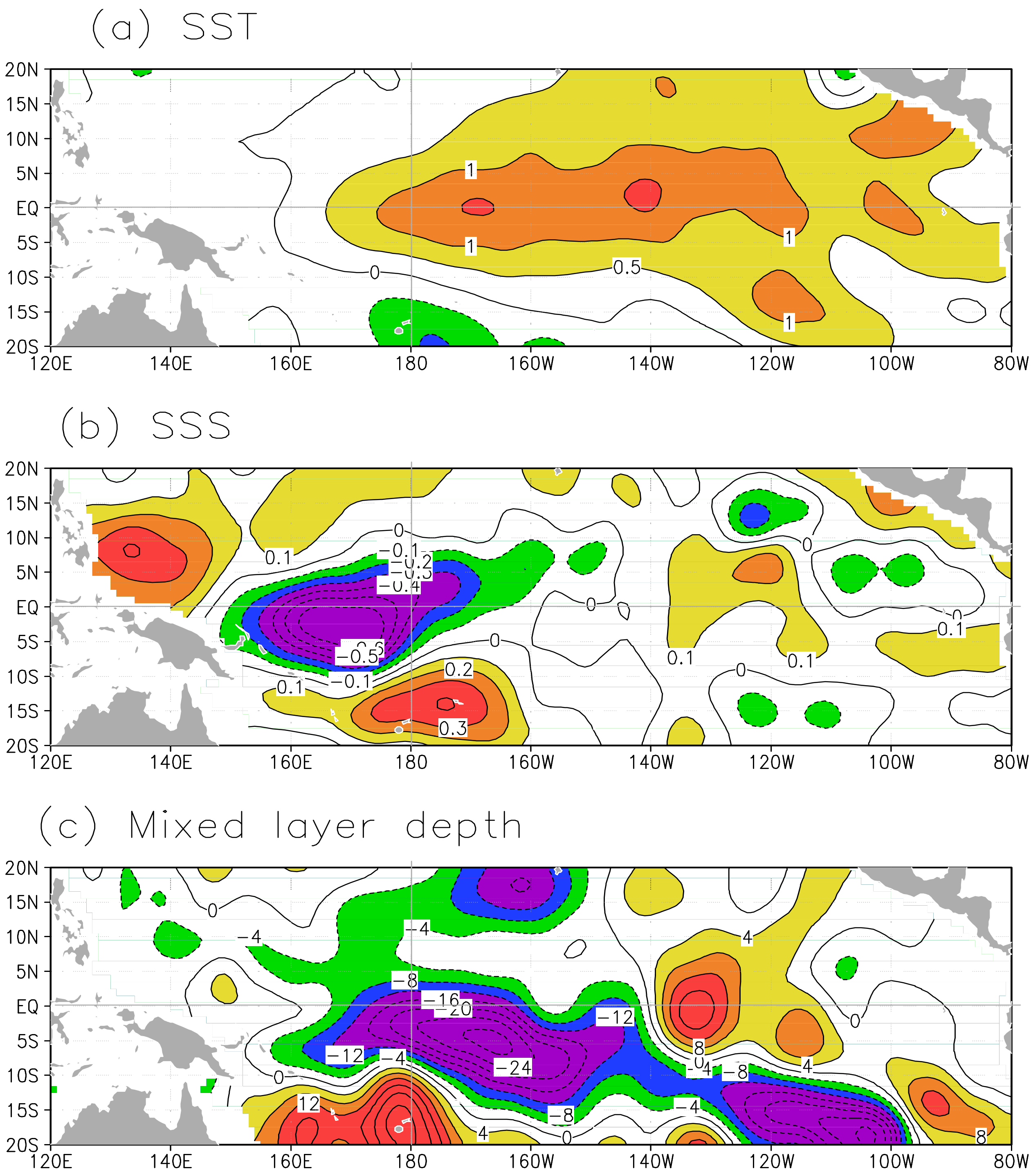


Fig. s9 Horizontal patterns of interannual anomaly fields observed from ARGO for El Niño conditions in October 2009: (a) SST, (b) SSS, and (c) MLD. The contour interval is 0.5°C in (a), 0.1 psu in (b), and 4 meters in (c).

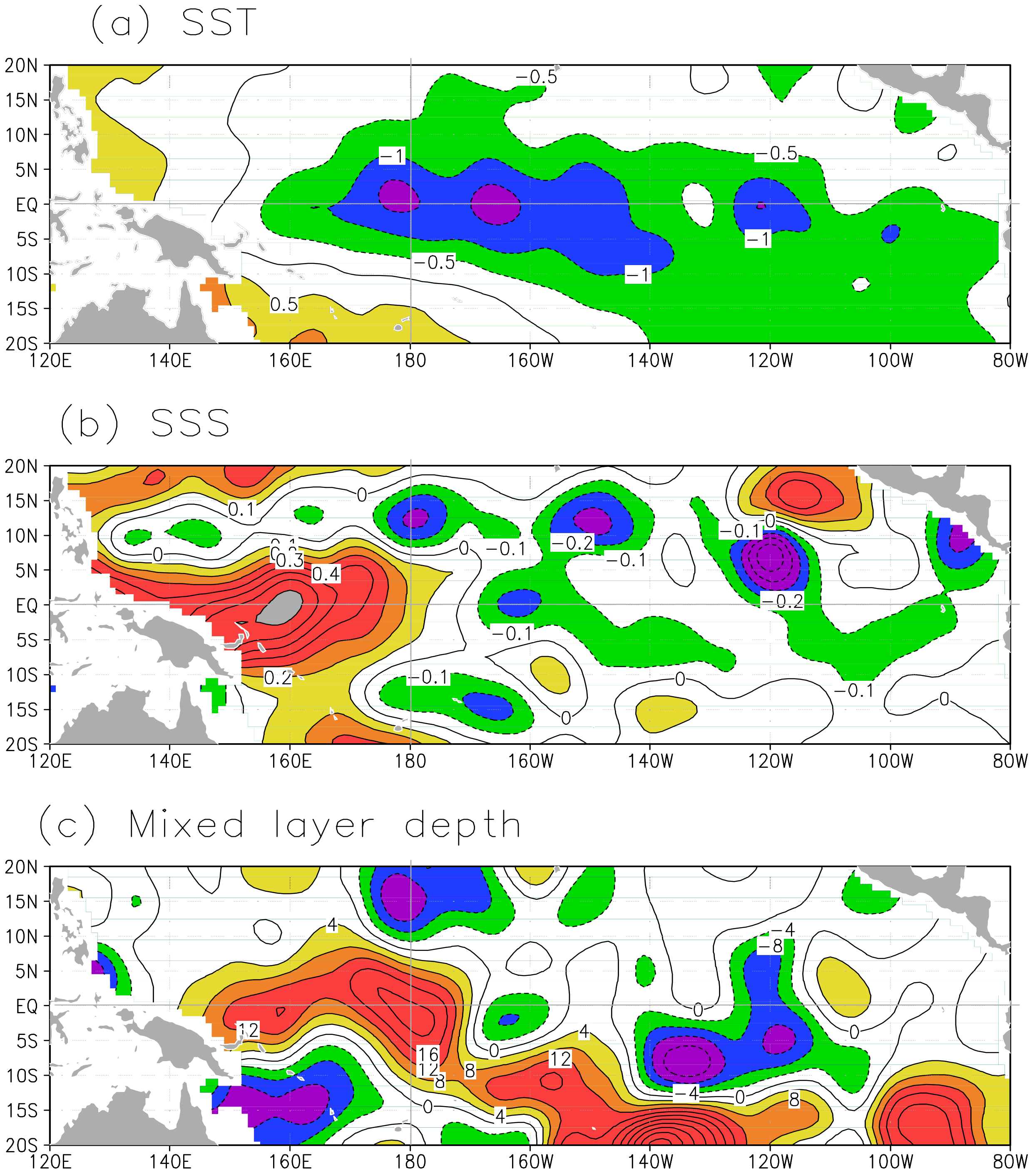


Fig. s10 The same as in Fig. s9 but for La Niña conditions in October 2010.

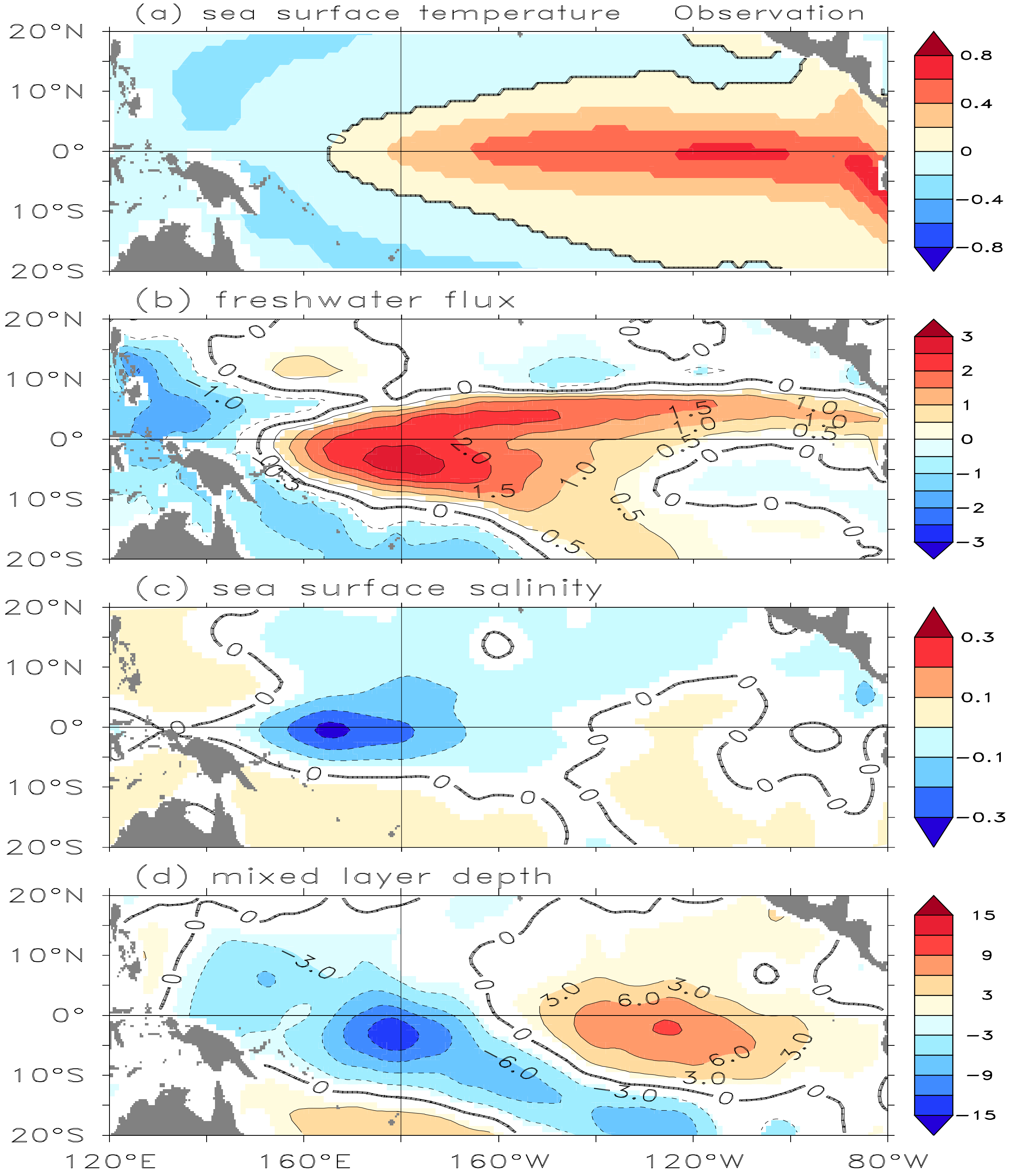


Fig. s11 The same as in Fig. s10 but for the analysis from observed ARGO data during 2005-2013 and freshwater flux during 1979-2008 (the Global Precipitation Climatology Project (GPCP) the Version-2 Analysis for precipitation (Adler et al. 2003) and OAflux for evaporation (Yu and Weller 2007)).

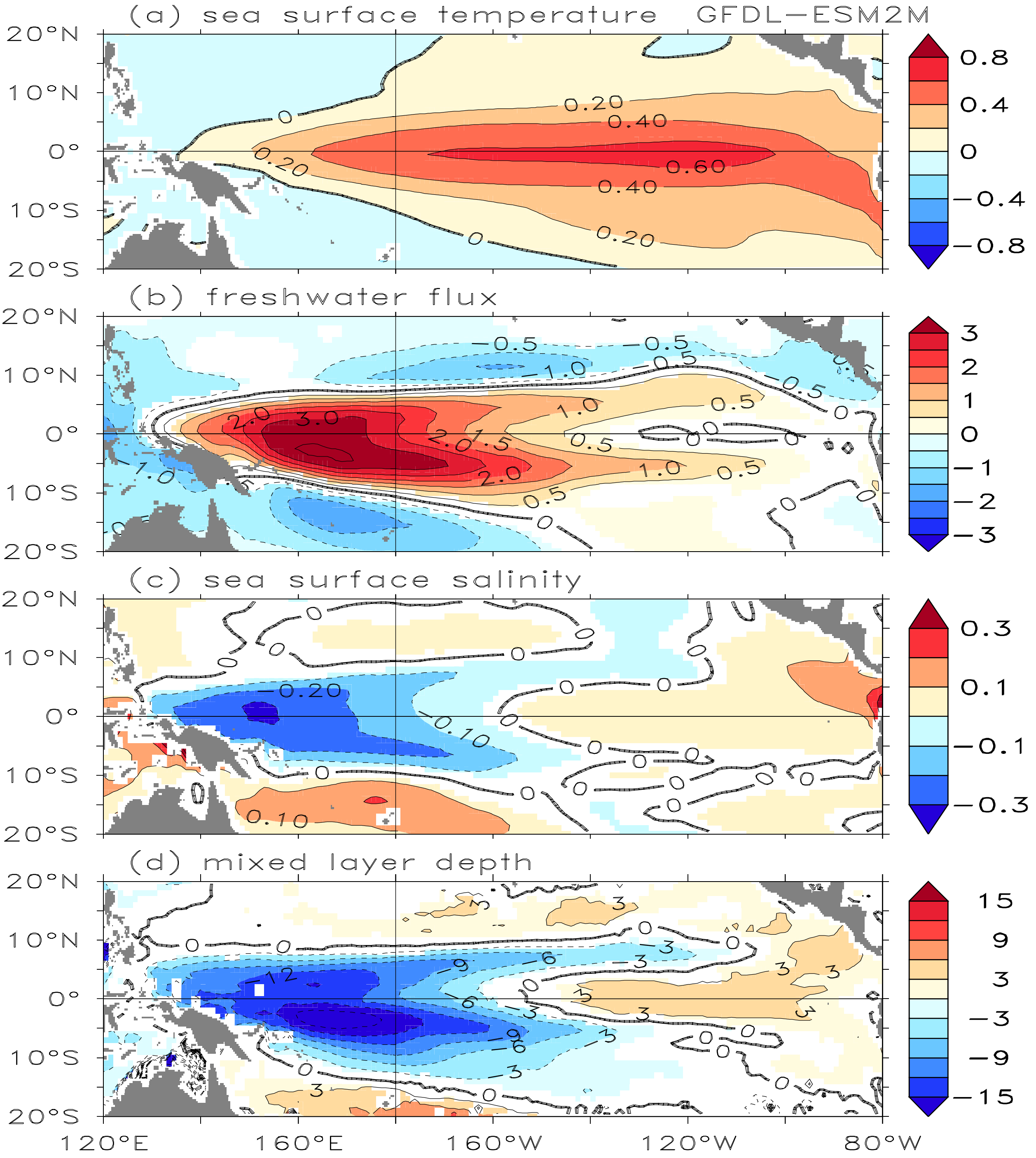


Fig. s12 The same as in Fig. s10 but for the analysis from the GFDL-ESM2 simulation. We make use of pre-industrial control (Pi-control) scenario runs; data from the last 100 years are selected.

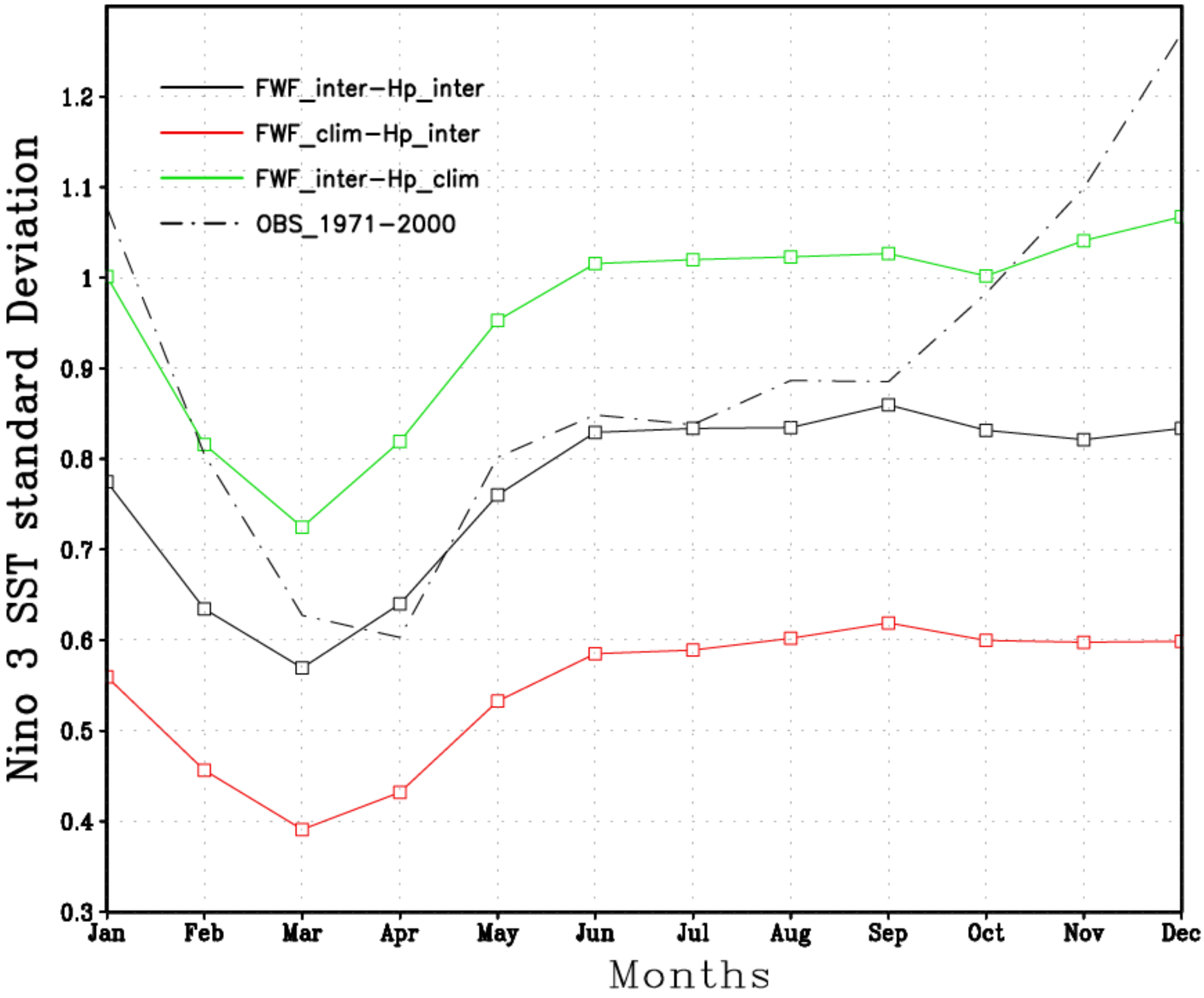


Fig. s13 Simulated and observed standard deviations of the Niño 3 SST anomalies (°C) as a function of the calendar month. The HCM-based simulations are calculated from model year 24 to 54 and the observation is calculated from year 1971 to 2000.

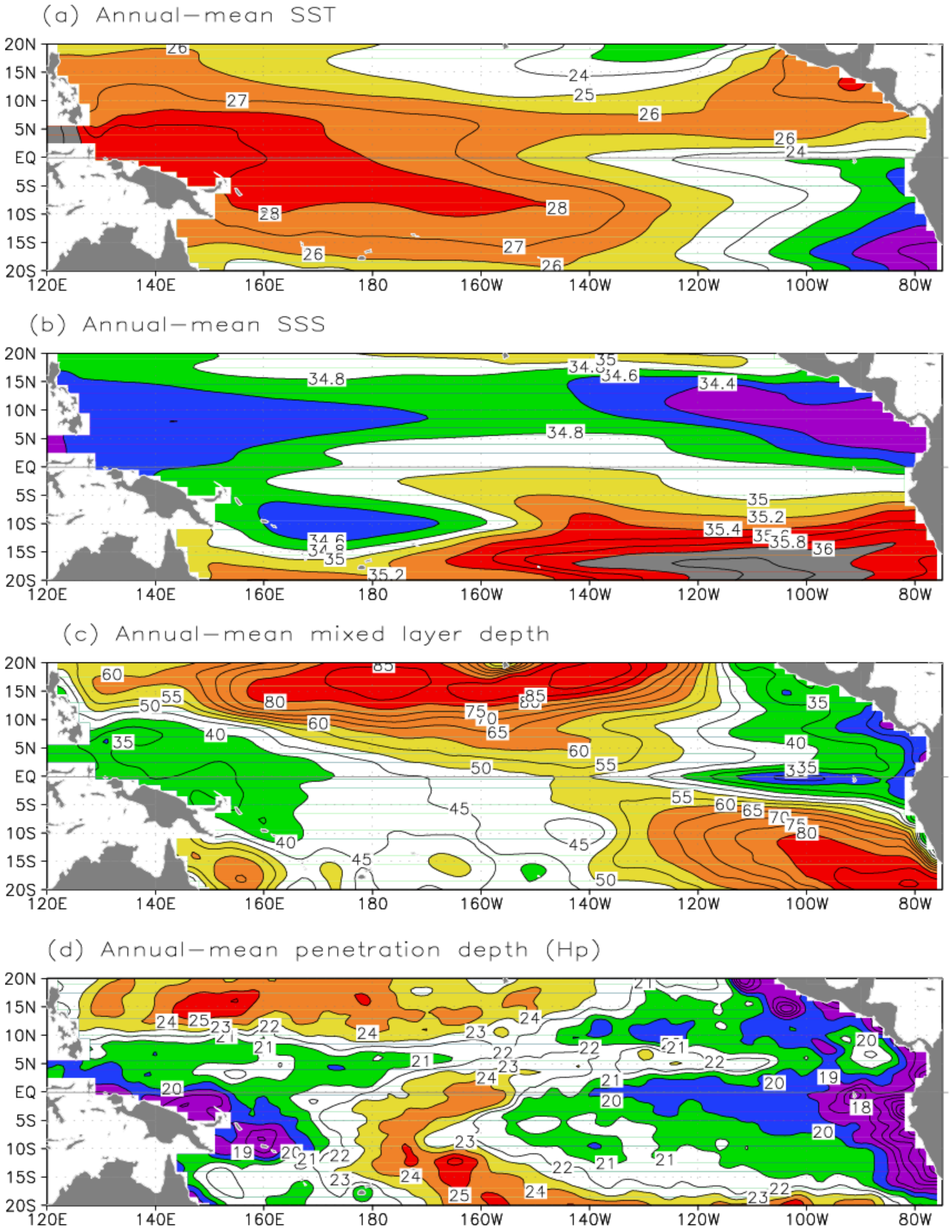


Fig. s14 Horizontal distributions of annual-mean fields simulated from the $FWF_{inter}$-$OBH_{inter}$ run for (a) SST, (b) SSS, (c) the mixed layer depth (MLD), and (d) the penetration depth ($H_p$). The contour interval is 1 °C for SST, 0.1 psu for SSS, 5 m for MLD, and 1 m for $H_p$.

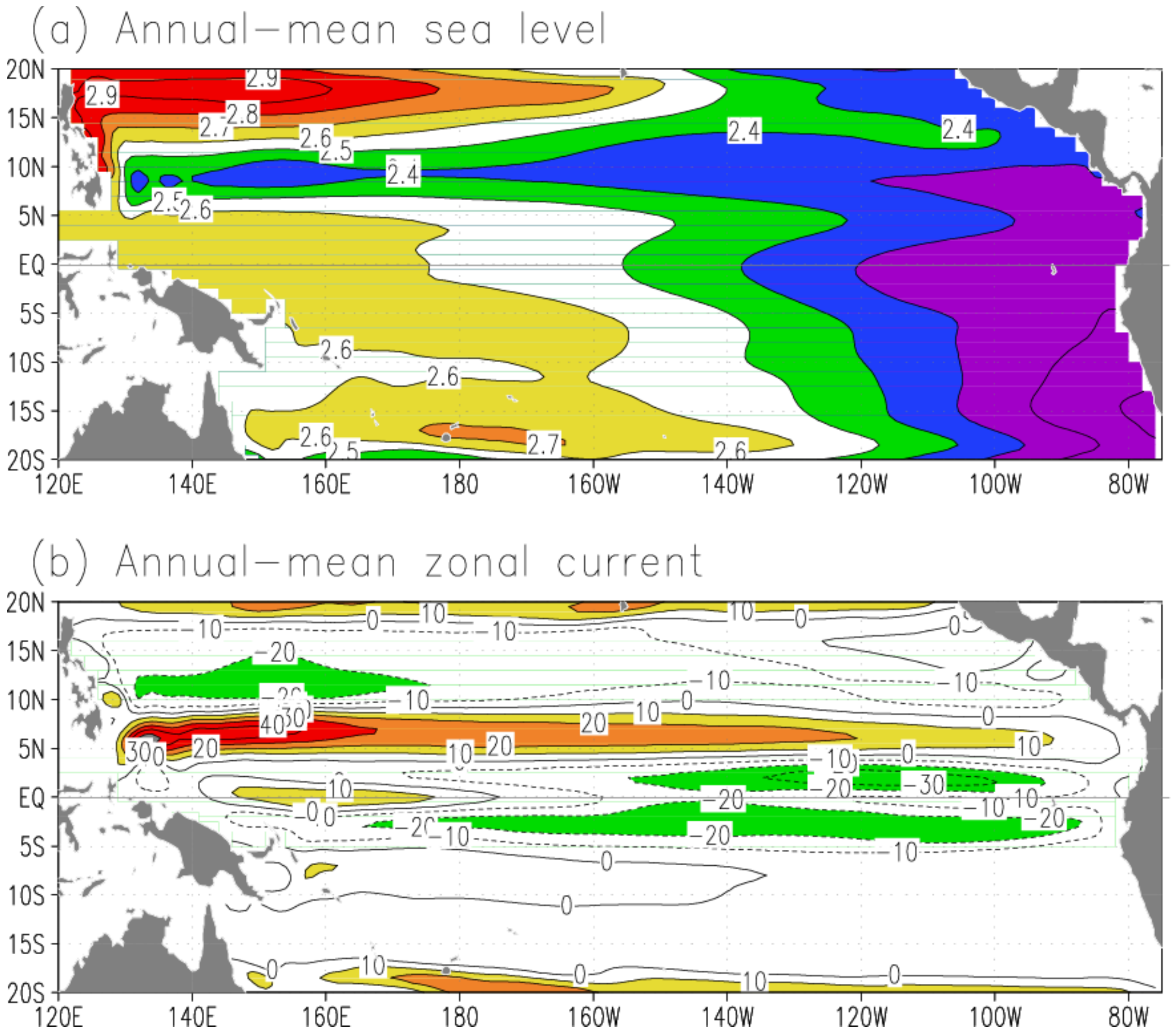


Fig. s15 Horizontal distributions of annual-mean fields simulated from the $FWF_{inter}$-$OBH_{inter}$ run for (a) sea level (SL) and (b) surface zonal current. The contour interval is 0.1 m in (a) and 10 cm $s^{-1}$ in (b).

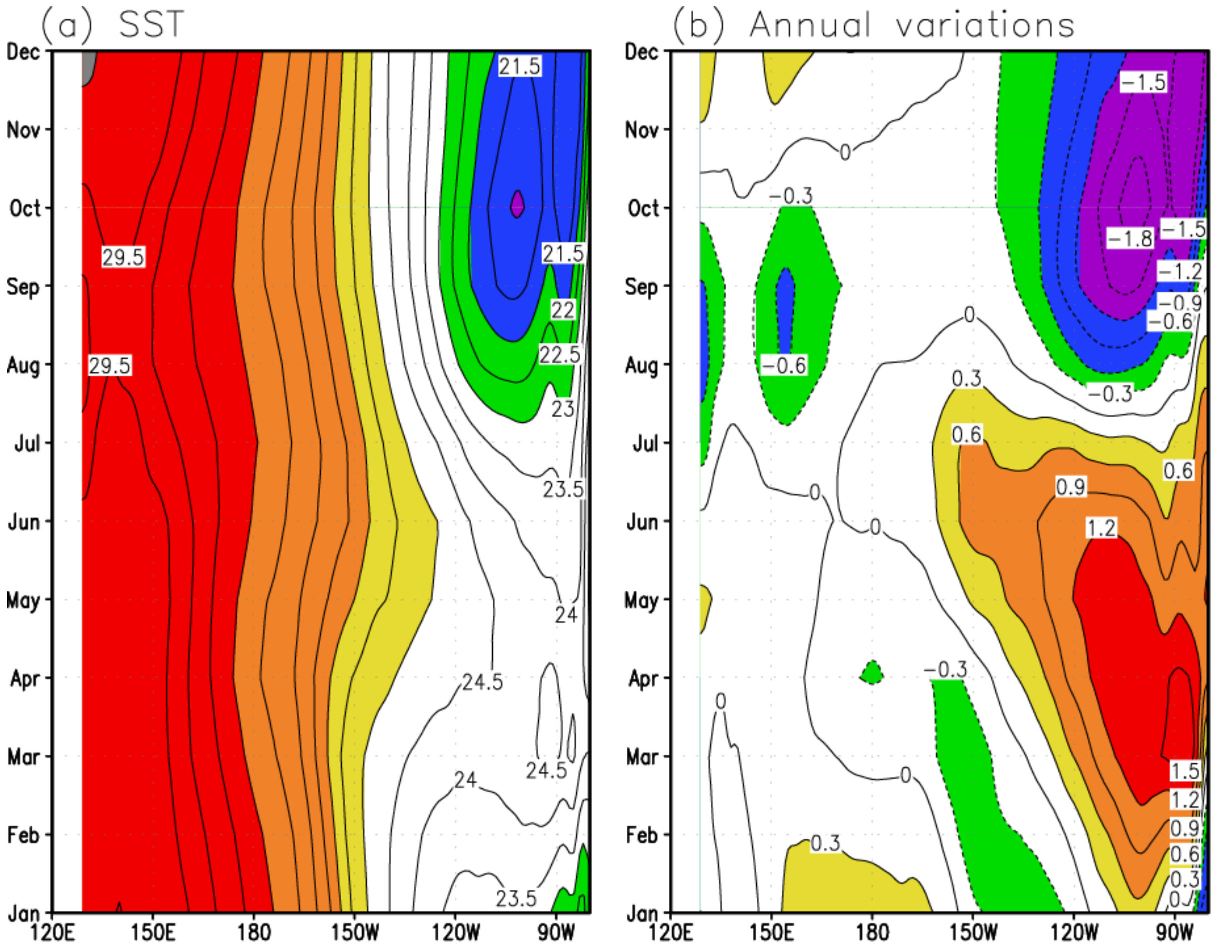


Fig. s16 The SST seasonal cycle (a) and annual variations (relative to its annual mean; b) along the equator simulated from the $FWF_{inter}$-$OBH_{inter}$ run. The contour interval is 0.5°C in (a) and 0.3°C in (b).

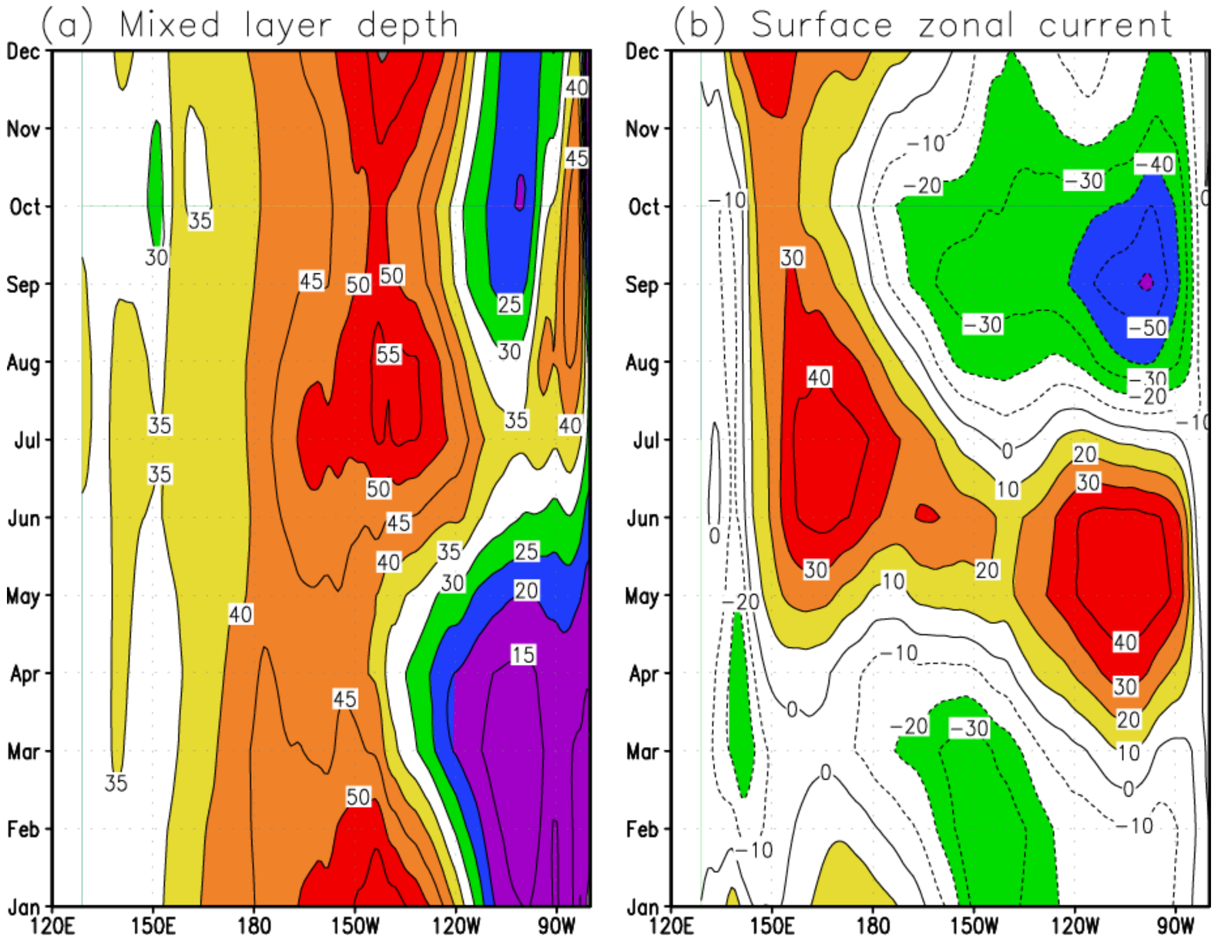


Fig. s17 The seasonal cycles along the equator for (a) MLD and (b) surface zonal currents simulated from the $FWF_{inter}$-$OBH_{inter}$ run. The contour interval is 5 m in (a) and 10 cm s$^{-1}$ in (b).

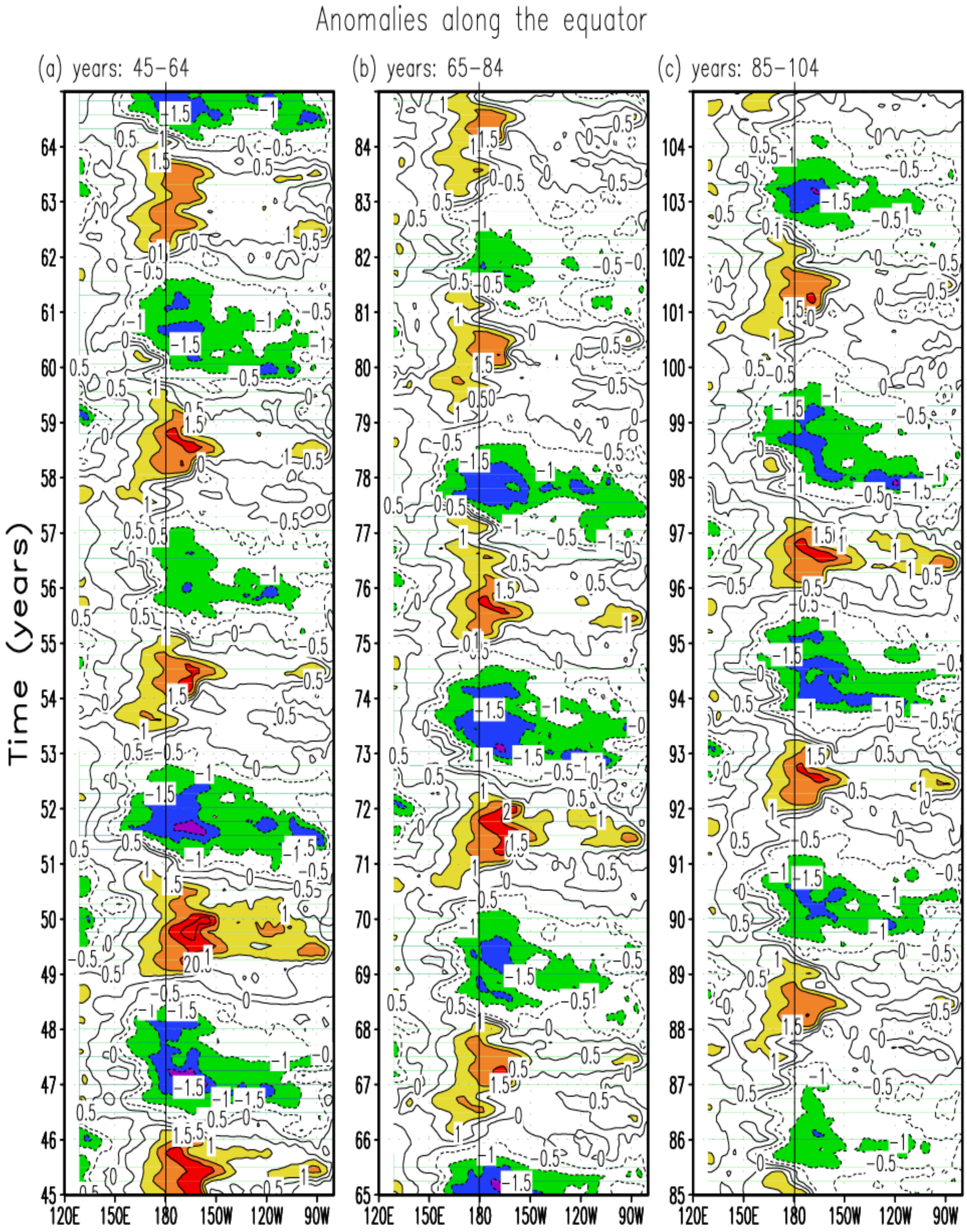


Fig. s18 Interannual SST anomalies along the equator simulated from the $FWF_{inter}$-$OBH_{inter}$ run. The HCM was integrated for more than 100 years and the plotting is shown for model year 45 to 104. The contour interval is 0.5 °C.

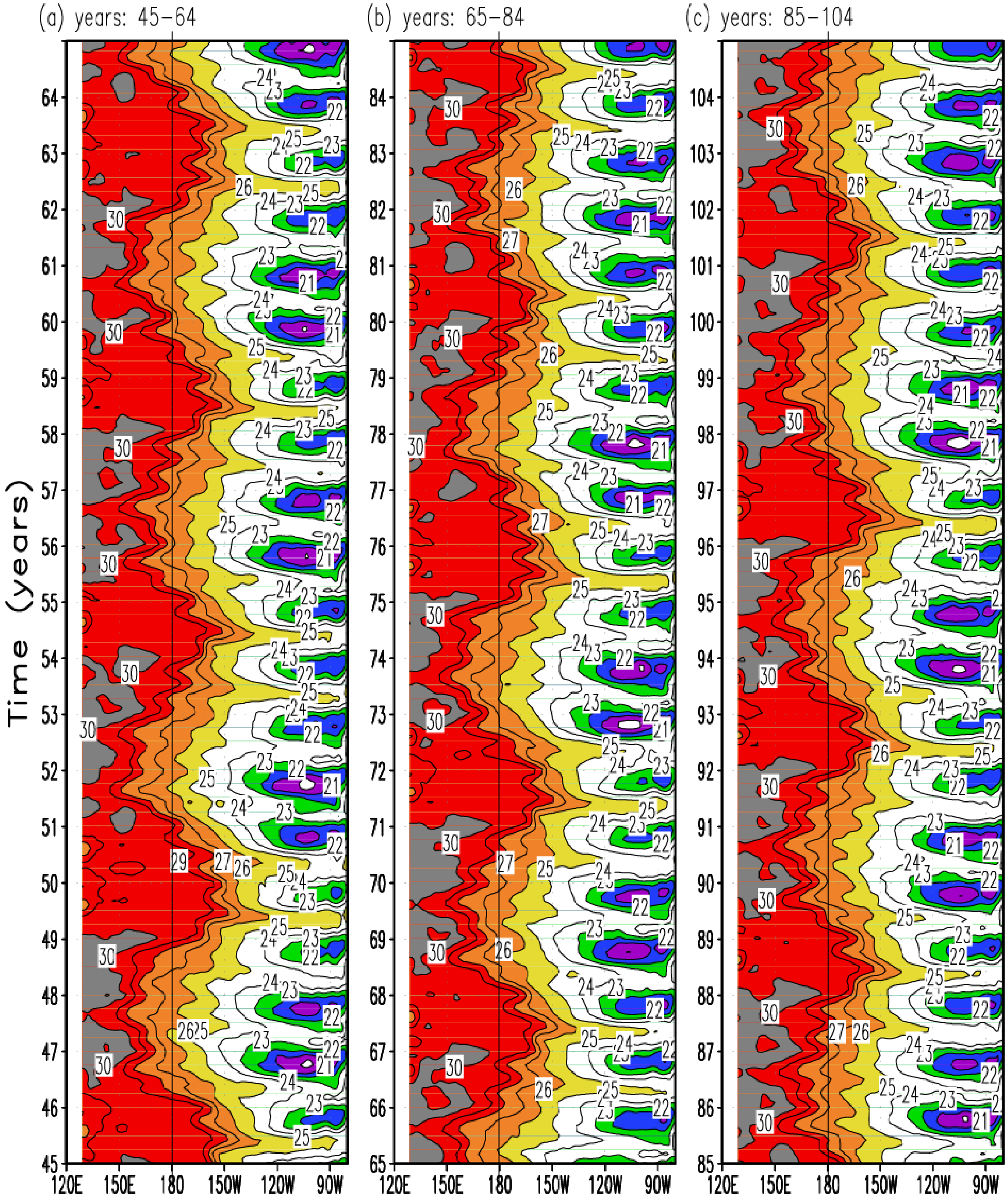


Fig. s19 The same as in Fig. s18 but for the total SST fields. The contour interval is 1 °C.

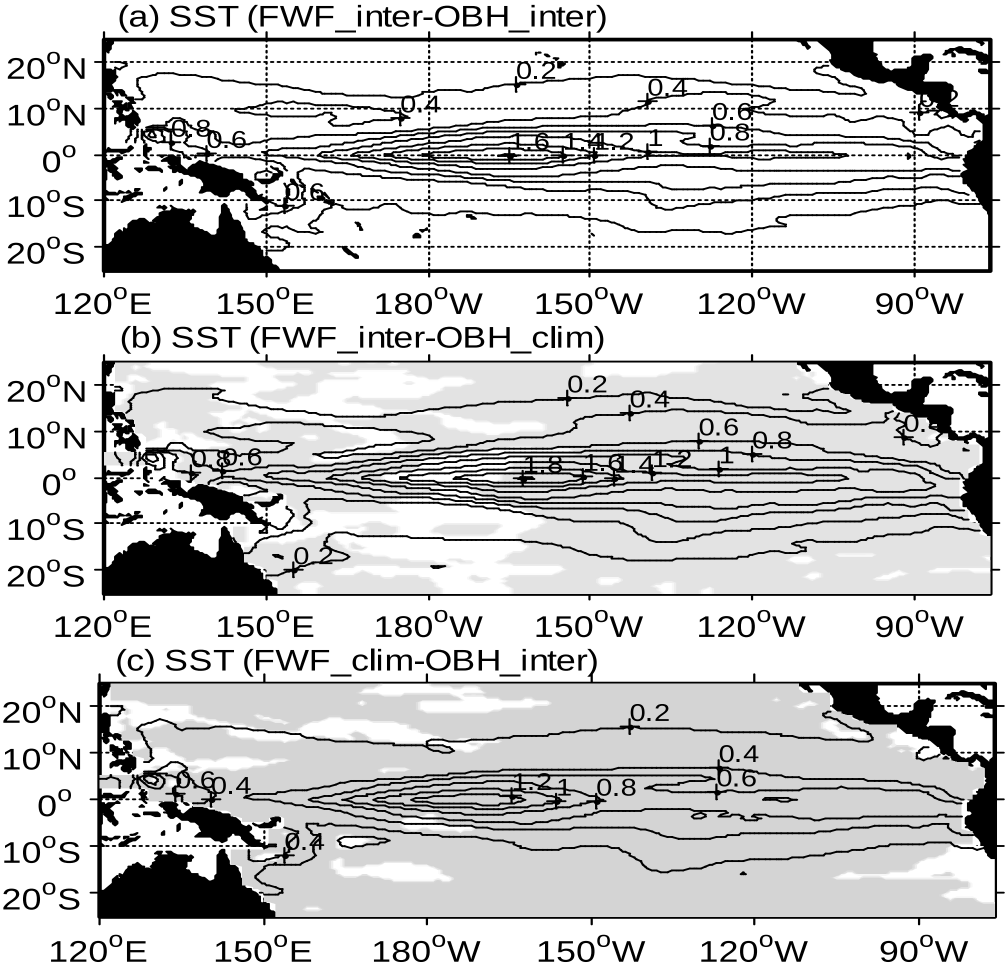


**Fig. s20** Horizontal distributions of the standard deviations for interannual SST variabilities calculated from different HCM experiments: (a) $FWF_{inter}$-$OBH_{inter}$; (b) $FWF_{inter}$-$OBH_{clim}$; (c) $FWF_{clim}$-$OBH_{inter}$. Additionally, to test if the changes in interannual SST variabilities between $FWF_{inter}$-$OBH_{inter}$ and $FWF_{inter}$-$OBH_{clim}$, and between $FWF_{inter}$-$OBH_{inter}$ and $FWF_{clim}$-$OBH_{inter}$ are significant, we apply an F-test to these interannual SST anomaly fields. The stippling regions in (b) and (c) indicate that the standard deviations in the $FWF_{inter}$-$OBH_{clim}$ and $FWF_{clim}$-$OBH_{inter}$ simulations are significantly different from those in the $FWF_{inter}$-$OBH_{inter}$ simulation. The contour interval is 0.2 °C.
